# 3D bio-screen printing for high-throughput production of scaffolds for meat alternatives

**DOI:** 10.1038/s41538-026-00853-0

**Published:** 2026-05-13

**Authors:** Robin Maatz, Philipp Karnop, Ryan Sylvia, Thomas Herget, Andreas Blaeser

**Affiliations:** 1 Institute for BioMedical Printing Technology, TU Darmstadt, Darmstadt, Germany; 2 Merck KGaA, Darmstadt, Germany; 3 Centre for Synthetic Biology, TU Darmstadt, Darmstadt, Germany

**Keywords:** Biotechnology, Engineering, Materials science

## Abstract

Increasing the production of structured cultivated meat (CM) remains a major challenge. Many current 3D printing and bioprinting technologies lack the necessary throughput, material compatibility, and cytocompatibility to produce realistic meat alternatives. In this study, screen printing, a traditional high-throughput printing technology, was investigated for the first time for the application of meat alternatives. The new process, 3D bio-screen printing (3D-BSP), presents a biomanufacturing process that uses edible materials (e.g., plant proteins) to produce meat-like structures with high resolution (0.1 mm). The suitability of edible inks for the 3D-BSP process was investigated on the basis of soy protein isolate (SPI) rheologically (flow index value < 0.4) and in terms of printability. In order to increase the protein content in edible inks while maintaining processability, an approach based on reducing agents was investigated. Sodium sulphite served as a demonstration model and resulted in a high protein content (>20 wt%) in SPI, while maintaining the flow properties suitable for processing protein-rich inks. To test the suitability as scaffolding technology for hybrid cultivated meat, meat-like scaffold structures were printed with C2C12 myoblasts differentiated on them. The printability of the structures was high in the resolution range investigated from 0.1 mm to 1 mm and supported 2D and 3D myoblast cultures (64% actin coverage and myotube formation). Ultimately, a marbled prototype was produced whose thickness could be increased by a stacking approach (>0.5cm). The texture profile (e.g., chewiness) of stacked and printed scaffolds was comparable to that of conventional meat. Upon successful transfer of the process parameters determined here to industrial screen printing machines, production rates of >100 kg/h could be achieved with one machine in the future. 3D-BSP offers a practical and cost-effective perspective for the mass production of structured meat in industrial quantities using screen printing technology. It could remove technical and economic barriers in this area and bring us closer to the commercial production of high-quality meat alternatives.

## Introduction

Cultivated meat (CM) represents genuine meat produced under laboratory conditions that can serve as a high-quality protein source while preserving global resources and animal welfare^1^. Unstructured product types, such as minced meat, are easier to produce and are therefore likely to be first on the market. In comparison, structured types, such as steaks, require scaffolding and bioprocessing technologies to create the more complex texture^2,3^. However, they make up an equal proportion of the market share and will play a crucial role in consumer acceptance of CM. The texture of structured CM is dependent on the 3D architecture of meat, which represents the cross-section of a muscle^4^. The muscle is composed of various cell types, including muscle fibres, fat cells, and connective tissue cells^5–8^. Skeletal muscle fibres develop from the fusion of myoblasts along a common axis while being embedded in a dense extracellular matrix (ECM) network called the connective tissue, in which collagen type I is the major structural protein.

Hybrid cultivated meat (HyCM) represents an early product type and consists, along with animal cells, of edible scaffolds and additives^9–11^. HyCM can increase the texture and nutritional content of CM while reducing costs for the consumer^12–14^. Among other biofabrication technologies, 3D printing combined with cell seeding and 3D bioprinting is explored for the manufacturing of scaffolds for HyCM^14–18^. The scaffolds must provide structural cues to support cell proliferation and differentiation while increasing the texture and nutritional value. Plant- or fungus-derived materials^9–11^, insect-derived^19^, as well as synthetic ECM molecules^20–22^ are currently examined for this purpose. Soy protein isolate (SPI) has shown its suitability as scaffold material^14,16,23,24^. In addition, 3D printing of SPI-based materials was explored to produce scaffolds with more biomimetic, meat-like structures^18,25^. However, low SPI contents were used (<5%), probably due to high viscosity and pre-printing gel formation, respectively, under specific conditions (i.e., solvent, pH, temperature, salts)^26^. 3D printing technologies used in this field require low initial viscosity to print at high resolution. Even an increase in the nozzle diameter can lead to a considerable loss of printing resolution^27,28^. Gel formation can be avoided through the usage of reducing reagents, such as Na_2_SO_3_ or cysteine, which prevent covalent disulfide bond formation^26,29,30^. To date, however, reducing agents have not been investigated for the additive manufacturing of CM or HyCM.

**Fig. 1:**
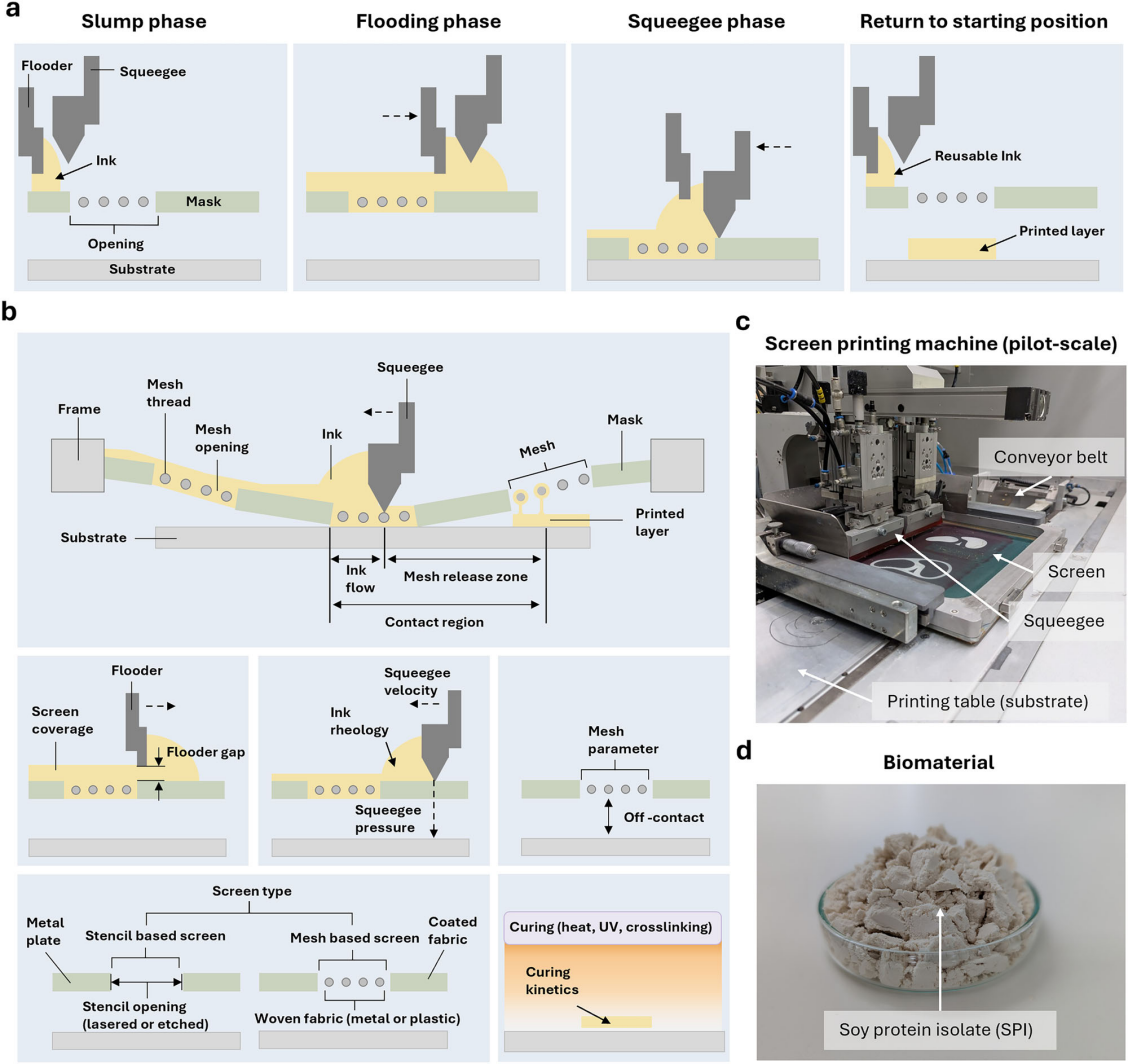
3D bio-screen printing process based on industrial screen printing. **a** Schematic overview of the three main screen printing steps. **b** Principal of screen printing parameter. **c** In-house screen printing machine on a pilot scale. **d** Soy protein isolate as a representative material for the formulation of inks for the production of plant-based scaffolds of hybrid cultivated meat.

The fundamental principle of screen printing is that ink is transferred through a screen onto a substrate in a specific two-dimensional pattern. The printing pattern is determined by covering the screen with a mask made of an impermeable material, which conceals the part that is not to be printed. A screen is composed of threads woven into a mesh that forms openings which can be perceived as pixels. The printing parameters are primarily dependent on the thread measures (diameter) and count (threads per area). Both the thread diameter and thread count have a direct effect on the thickness of the printed layers and the size of the mesh openings. This parameter, in turn, influences the resolution of the printed image. The printing process itself can be divided into three phases in which different shear rates of up to 10,000 s^−^^1^ occur (Fig. 1a). In order to fully exploit the rheological interactions and ensure efficient material transfer, inks with a high initial shear viscosity (> 100 Pa^.^s) and strong shear thinning behaviour (viscosity decreases with higher shear rates) are used for screen printing. In addition, a suitable yield stress (a minimum stress that is required to make the inks flow) is important. It must allow the initial flow of the material during flooding, prevent the ink from bleeding out of the mesh, and ensure stability after transfer to the substrate^31–33^ (Fig. 1a). Therefore, the high range of shear rates must be considered in the development of printable inks. (1) In the first stage, ink is applied to a screen, and a flooder pushes the ink over the screen as the ink flows into the mesh opening. At this stage, the viscosity of the ink is reduced. (2) Next, a squeegee makes contact between the ink-filled mesh and the substrate, whereupon the mesh is dragged from the ink. This is where the highest shear rates occur (3). The shear ends, and the material recovers, causing the viscosity to increase again.

Screen printing has been used for decades in the industrial mass production across different sectors. Its high production capacity and free-form design make it applicable to a wide range of industrial products, such as textiles, electronics^34–37^ and food^38^. Scalability is achieved through different parameters such as screen size (printing area), number of printing stations, screen type (mesh or stencil-based), number of printing tables, or substrate handling (i.e., conveyor belt) (Fig. 1a, b, c and Supplementary Fig. 1a, b).

Although screen printing was originally developed for two-dimensional printing, it is now being investigated for mass-producing three-dimensional structures^34,35,39–41^. It has been tested for the production of pharmaceutical tablets^41^ as well as for metal components such as complex-shaped titanium parts^35^. Further, hand-operated 2D-screen printing was explored for printing cellular tissues^39,40^. The productivity of 3D screen printing is comparable to that of injection moulding. This is the crucial advantage over other additive manufacturing techniques: For both technologies, the costs per part decrease with the number of produced parts, making it at large-scale more economically compared to conventional 3D printing^41–43^.

**Fig. 2:**
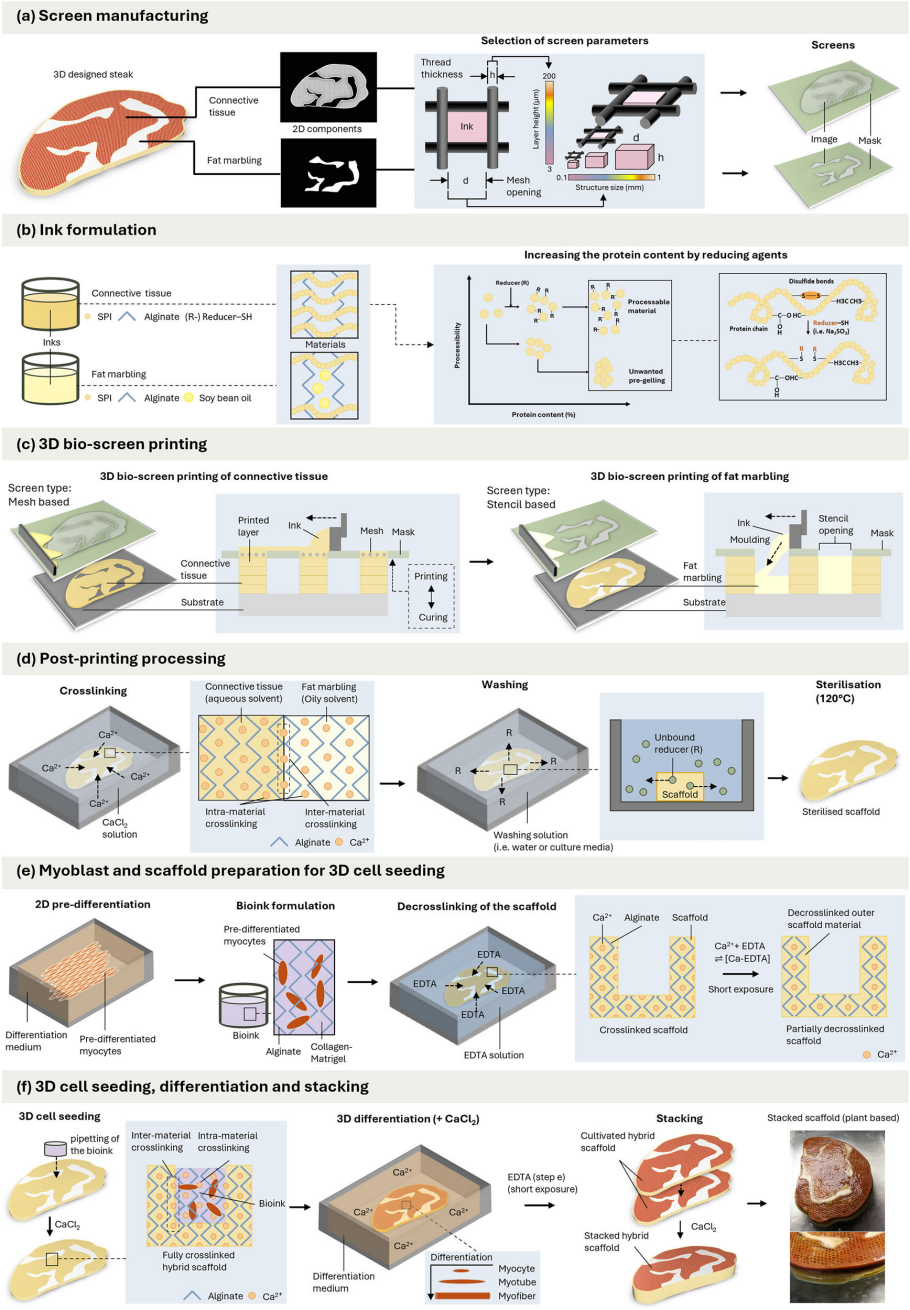
3D bio-screen printing for the production of cultivated meat. Process flow of the here developed 3D bio-screen printing process with schematic description of the individual process steps.

In this study, we aim to exploit the advantages of 3D screen printing for 3D bioprinting purposed for the first time and demonstrate this using the example of high-throughput production of structured CM. Through multiple scaling steps (Supplementary Fig. 1a), production rates of over 1000 kg/h could be achieved if the scaling parameters of traditional industrial screen printing are applicable to the 3D bio-screen printing process proposed here. This depends, among others, on the compatibility of the ink with larger screen sizes and screen parameters, the post-treatment of the printed layer, such as heating, and the compatibility with substrates used in high-speed machines. If applicable, the throughput could be comparable to established technologies for plant-based products, such as high moisture extrusion (Supplementary Fig. 1b). Furthermore, at constant throughput, the complexity of the printed constructs in traditional screen printing remains comparable to that of conventional 3D extrusion printing, achieving resolutions below 100 µm^40^. In contrast to high-moisture extrusion, which requires elevated temperatures and pressure and may compromise the nutritional quality of the final product, the printing step in The 3D bio-screen printing (3D-BSP) operates at ambient temperature. This gentle deposition method enables the formation of meat-like structures while better preserving the nutritional integrity of the material. The 3D-BSP process comprises seven steps: (i) the digital design of the print image and manufacturing of a scaffold or tissue (ii) selection of 3D-BSP parameters and screen manufacturing (iii) development of scaffold materials and bioinks (iv) 3D-BSP of scaffolds and (v) post-processing of the scaffolds, such as washing and crosslinking (vi) 2D/3D cell seeding or 3D bioprinting with the scaffolds (vii) cultivation of the (hybrid) CM construct (Fig. 2). The results achieved demonstrate the fundamental transferability of the method to the field of biofabrication of meat alternatives and beyond. It could be an important step towards bioprinting becoming an industrializable process that, in the future, could support not only the field of CM, but also other industries such as the scaled-up bioproduction of in vitro tissue models.

## Results

### Protein-based hydrogels are suitable for 3D-BSP

**Fig. 3:**
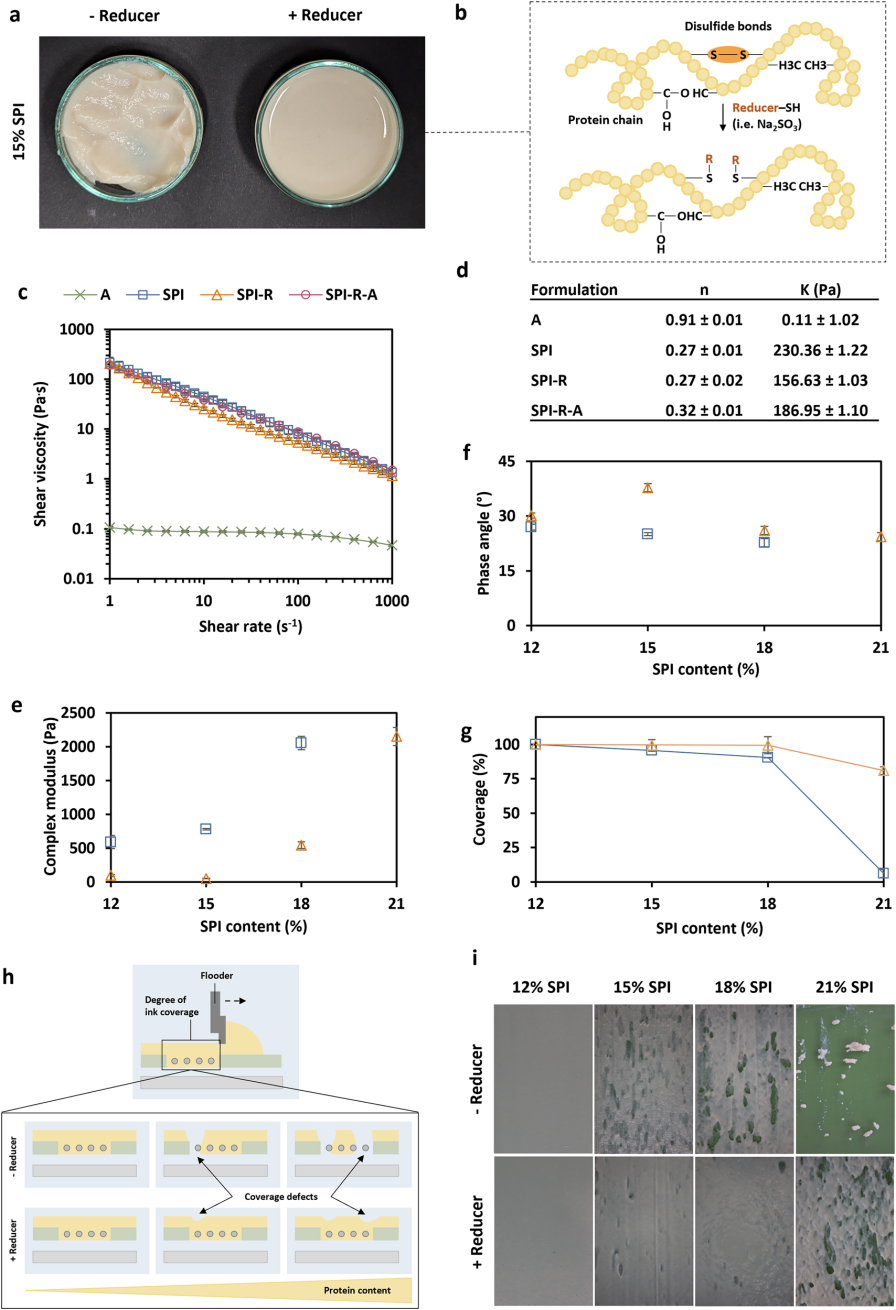
Development of SPI-based hydrogels for scaffolding through 3D bio-screen printing. **a** Representative image of soy protein isolate without (left) and with (right) reducer (sodium sulphite). **b** Schematic illustration of the reduction mechanism of sulphite groups. **c** Shear viscosity curves of alginate (A), soy protein isolate (SPI), reduced (sodium sulphite) SPI (SPI-R) and reduced SPI with alginate (SPI-R-A). **d** Mean and standard error (SE) of consistency factor (K) and flow index value (*n*), calculated from shear stress curves. **e** Complex modulus (G*) and **f** Phase angle (φ) of different SPI content (12– 21%) with (orange) and without reducer (blue, sodium sulphite). **g** Degree of coverage over SPI content with (orange) or without reducer (blue, sodium sulphite). Data points represent the mean, and error bars represent the standard error (SE) with *n* = 3. Due to the too high strength, 21% SPI without a reducer could not be measured rheologically. **h** Schematic representation of the reducer-dependent degree of coverage during the flooding step. **i** Representative photographic images of different SPI content (12% – 21%) flooded over substrate without (upper) or with (lower) sodium sulphite.

Soy proteins are the most used plant protein to produce meat alternatives, including hybrid cultivated meat. Therefore, we have chosen soy protein isolate (SPI) as a demonstration material for the development of the 3D bio-screen printing process (Fig. 1b). However, a more versatile selection of materials must be available in the future that are more suitable in terms of sustainability in material production and flavour. 15% SPI exhibited a high viscosity that was greater than 200 Pa.s (Fig. 3c). In addition, it had a strong shear thinning behaviour, indicated by a low flow index value (n, Fig. 3d). In order to achieve high protein contents (> 20 wt%) an energy-efficient approach was investigated through the use of reducing agents. We tested the utilisation of reducing agents on the example of Na_2_SO_3_ for the preparation of printable soy

protein isolate (SPI) hydrogels with high protein contents (Fig. 3a). By the addition of Na_2_SO_3_ the shear viscosity (η) of 15% SPI hydrogels was slightly decreased by 11% (*P* = 0.05242; Fig. 3c). In addition, no significant difference was observed in the shear thinning behaviour, as indicated by similar flow index values (*n*, *P* = 0.99965; Fig. 3d).

Reduced SPI hydrogels (SPI-R) were significantly less stiff (complex modulus G*) and showed lower elastic proportions (Phase angle φ) compared to non-reduced SPI hydrogels (G*, *P* = 0.00095; φ, *P* = 0.00503; Fig. 3e, f). By adding reducer, 12% SPI experienced a decrease in stiffness of 6.1-fold (*P* = 0.00053; Figs. 3e) and 1-fold in the elastic proportion (*P* = 0.00143; Fig. 3f). Increasing the SPI content from 12% to 18% caused a 3.5-fold change of higher stiffness without reducer and 5.6-fold with reducer (*P* = 0.000016; *P* = 0.00175; Fig. 3e). In addition, the phase angle dropped for the non-reduced hydrogel from 27 ± 0.8° to 22.7 ± 1.1° and for the reduced from 30.1 ± 0.7° to 24.4 ± 1.1° (*P* = 0.0118; *P* = 0.01476), indicating an increase of the elastic proportion. Reversed to the behaviour without reducer, 15% SPI supplemented with sodium sulphite showed a lower elasticity (φ *P* = 0.0014) and stiffness (G* *P* = 0.64848) compared to 12% SPI-R (Fig. 3e,f).

Next, the effect of reducing agents on screen printing (flooding step, Fig. 3h) was examined (Fig. 3g, i). To do so, coverage efficiency was analysed as an indication of complete flooding. We observed a significant increase in coverage through the usage of reducing agents (*P* = 0.0033). Increasing the SPI content from 12% to 18% didn’t change the coverage significantly for reduced SPI, thus maintaining high coverage of 99% (*P* = 0.99507; Fig. 3g, i). In comparison, non-reduced SPI experienced a loss of 9% (*P* = 0.0354; Fig. 3g, i). Reduced SPI content of 21% showed a coverage of 81% compared to 12% (*P* = 0.00117), while non-reduced dropped to 6% (12% 21%, *P* = 0.000000011; Fig. 3g, i).

SPI-hydrogels were supplemented with alginate (SPI-R-A) as a binder to improve stability of textured multi-material scaffolds (i.e., muscle-, connective tissue-, and fat-mimicking layers). 1% alginate was found to increase the viscosity of 15% SPI-Reducer insignificantly by 9% (consistency factor K, P = 0.55479; Fig. 3c). As expected, alginate exhibited a nearly newtonian flow behaviour with *n* = 0.908 ± 0.007, which only slightly changed the strong shear thinning behaviour of the scaffold material from *n* = 0.272 ± 0.019 to *n* = 0.321 ± 0.012 (*P* = 0.01029; Fig. 3d).

### Meat-like structures can be realized through 3D-BSP

**Fig. 4:**
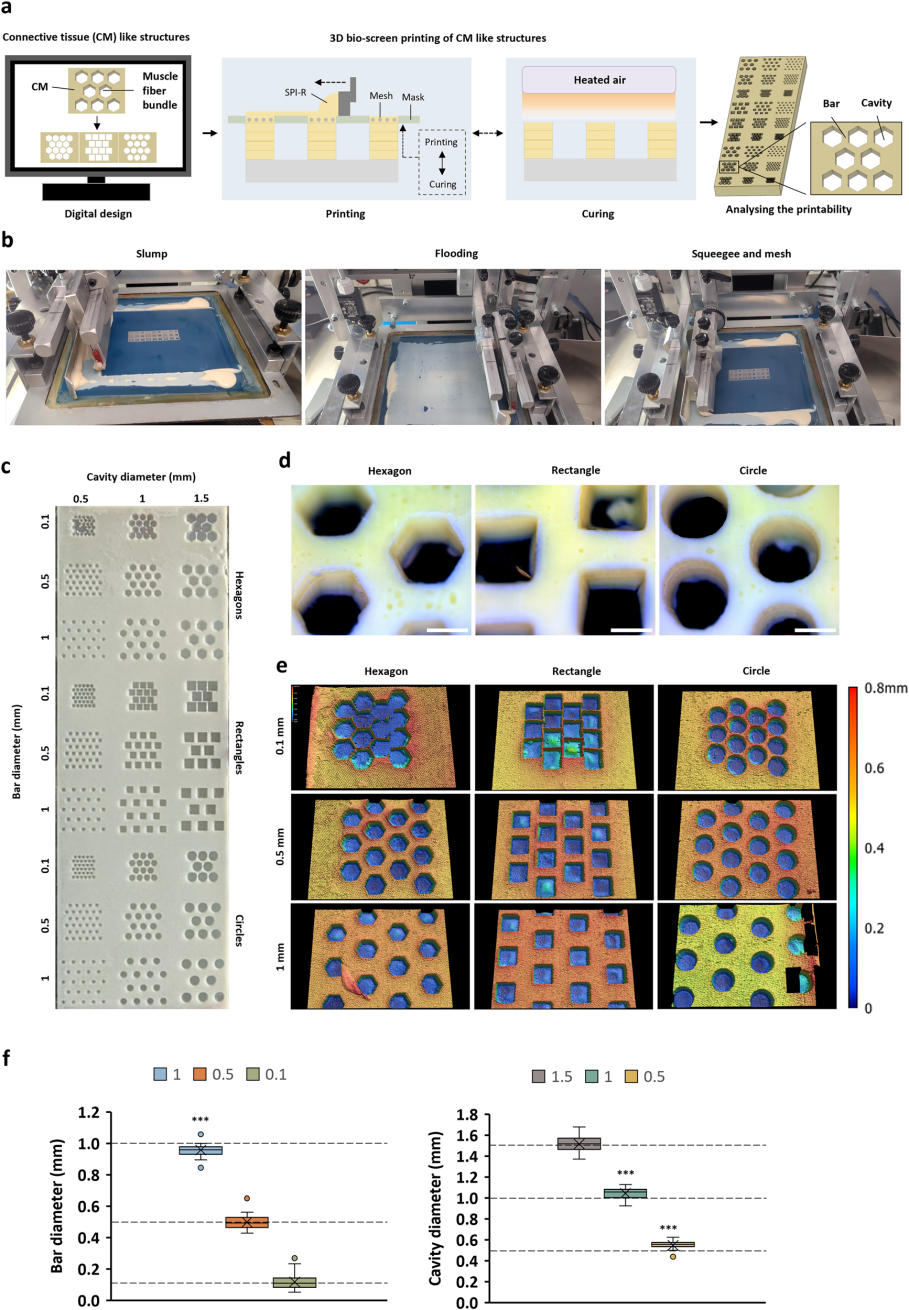
Analysis of 3D bio-screen printing of meat-like structures. **a** Schematic overview of the creation of connective tissue-like structures using the 3D bio-screen-printing process. **b** 3D bio-screen printing steps for the generation of connective-like tissue structures. **c** Photographic image of a printed construct consisting of connective tissue-like structures. **d** Microscopic images of printed connective tissue-like structures. Scale bar = 1000 µm. **e** 3D-profilometry images of the three different geometric forms (hexagon, rectangle and circle) with 1 mm inner diameter and varying bar-diameter: 0.1 mm (upper), 0.5 mm (middle) and 1 mm (lower). **f** Boxplots for absolute bar diameter (left) and absolute cavity diameter (right) calculated from the entirety of all structures with *n* = 27 for bar diameters and *n* = 54 for cavity diameters. Scattered lines represent the theoretical values of the digital design. The Wilcoxon test was used to compare the printed diameter with the theoretical diameter. The significance levels are indicated as follows: *p<0.05, **p<0.005, ***p<0.001.

We investigated 3D bio-screen printing (3D-BSP) as scaffolding technology by testing the printability of connective tissue-like structures (Fig. 4a). A construct was printed consisting of bar structures with a defined diameter (closest distance between two parallel cavities) (Fig. 4a,b). The bars formed three different geometric cavities (circle, rectangle, and hexagon) with defined inner diameter (Fig. 4c). The microscopic analysis of the printed 3D construct showed that shape fidelity was high with regard to the intended geometry (Fig. 4d, e).

The absolute deviation between the printed and theoretical bar structures was found to be within a small range of 0.046 mm–0.053 mm for all resolutions (Fig. 4f). Wider structures experienced a reduction (1 mm, −4.2%, *P* = 0.000412) and thinner structures an increase in diameter (0.1 mm, 19%, n.s; Fig. 4f). Normalization to the theoretical diameter also showed that higher resolution was more affected by deviation than lower resolution (Supplementary Fig. 2). As for the bar structures, cavity diameters showed equal consistency in deviation along the applied resolution range with 0.037 mm to 0.067 mm (Fig. 4f). Also, structures with higher resolution were more affected by absolute deviation compared to those with lower resolution (Supplementary Fig. 2).

### 3D bio-screen printed marbled scaffolds are compatible with food and cell processes

**Fig. 5:**
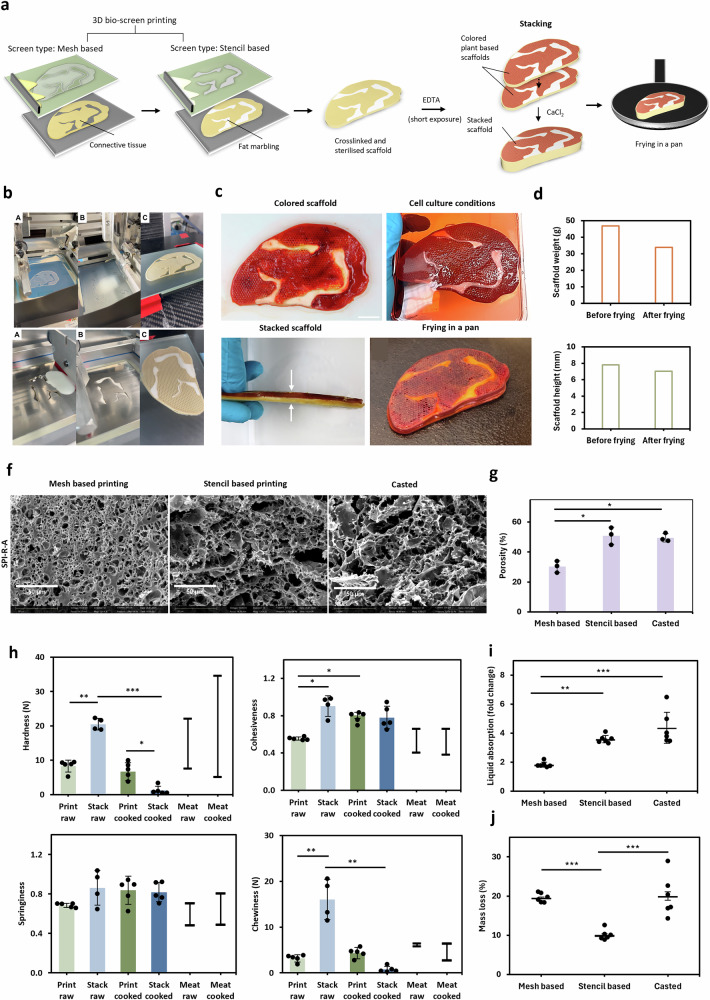
3D bio-screen printing and characterisation of plant-based scaffold. **a** Necessary process steps required to generate a steak-like scaffold from the overall process: (**c**) printing, (**d**) crosslinking, (**e**) re-crosslinking, and (**f**) stacking. **b** 3D bio-screen-printing steps for generation of a scaffold: Connective tissue (upper) and fat marbling (lower). **c** Post printing food processing steps of the scaffold. Scale bar = 2 cm**. d** Height and weight analysis of the scaffold before and after frying in a pan with *n* = 1. **f** Scanning electron microscopy (SEM) images show the microstructure of scaffolds printed either by mesh-based or stencil-based screen printing or cast as a control, and **g** quantified porosity measured from the SEM images. bars show the mean, and error bars show the standard deviation (SD), while single data points represent technical replicates, with *n* = 3. A two-way ANOVA and a post hoc Tukey test were used to compare the data across the three groups. The significance level is indicated as *p < 0.05. **h** A comparison of the textural profiles (hardness, cohesiveness, springiness and chewiness) of raw and cooked plant-based scaffolds was made. The scaffolds were either printed to the final height (print raw, print cooked), or printed and then assembled from multiple individual scaffolds to the final height (stack raw, stack cooked) using the SPI-A ink. Horizontal lines show the mean, and error bars show the standard deviation (SD), while single data points represent technical replicates with *n* = 4 for 'stack raw' and *n* = 5 for all others. A two-way ANOVA and a post-hoc Tukey test were used to compare the four groups' data. The significance levels are indicated as follows: *p < 0.05, **p < 0.005, ***p < 0.001. For comparison, three values for conventional raw and cooked meat were taken from the literature for each texture profile measurement parameter and shown as a range, with horizontal lines showing the minimum and maximum values. Literature: Hardness^76,77,79–82^, Cohesiveness^76–80,82^, Springiness^61,76,78–80,82^ and Chewiness^76–80,82^. **i** Liquid absorption and **j** mass loss for scaffolds printed with a mesh-based screen, a stencil-based screen or cast as a control using the SPI-A ink. Horizontal lines show the mean, and error bars show the standard deviation (SD), while single data points represent technical replicates with *n* = 6. A two-way ANOVA and a post hoc Tukey test were used to compare the data across the three groups. The significance levels are indicated as follows: **p < 0.005, ***p < 0.001.

We developed and characterised an in-line approach for advanced texturizing through fat marbling (Fig. 5a). First, a hybrid steak-like scaffold was designed that contained the main components of meat (connective tissue, fat, and muscle fibre bundles). Next, the plant-based connective tissue mimicking scaffold was printed using the SPI reducer alginate formulation (Fig. 5b and Supplementary Fig. 3a, b). For fat marbling, a plant-based biomaterial was printed within one strike into the scaffold, using a stencil-based screen (Fig. 5b). The fat substitute was developed on the basis of soy protein isolate and soybean oil. The material showed flow properties suitable for 3D-BSP: it exhibited a pseudoplastic behaviour under shear with the viscosity decreasing from 42.11 Pa.s to 0.11 Pa.s and recovered back to 42.52 Pa.s when shear was reduced (Supplementary Fig. 4c). After printing, the material remained stable without flowing out of the scaffold (Fig. 5b). The elastic proportion of the biomaterial dominated at the applied printing temperature (25 °C) indicated by a phase angle of 39.5°. (Supplementary Fig. 4a). Cooling from 45 °C–5 °C increased the elastic properties (phase angle) by 0.92-fold and the stiffness (complex modulus) by 2.2-fold (Supplementary Fig. 4a,b).

The compatibility of cell culture processing steps was verified on plant-based scaffolds (Fig. 5c). Maintenance under cell culture conditions showed dimensional stability during a 14-day period under cell cultivation conditions. A stacking approach was used, in which two individually printed scaffolds were stacked on top of each other. In addition to increasing the thickness of the final construct, this could be an approach to enable the stacking of multiple layers of living cells. This, in turn, could help circumvent the diffusion limit, which is critical when culturing tissue blocks in the centimetre range. Furthermore, stacking could be a useful strategy for scaling, as it provides an alternative to 3D printing the final height. Stacking involved several steps. First, each scaffold was cross-linked in CaCl_2_ to ensure stability under alginate cultivation conditions. After sterilization at 120 °C (autoclaving) and an incubation period in cell culture media, the scaffolds were briefly held in EDTA (10 seconds) to reverse the cross-linking of the alginate on the surface of the scaffolds. Finally, the scaffolds were stacked on top of each other, followed by a second CaCl_2_ cross-linking step to fuse the two scaffolds together (Figs. 2, 5c). The final height of the stacked scaffold was 7.72 mm with a weight of 46.86 g. Next, the compatibility of various food processing steps with the plant-based scaffold was demonstrated (Fig. 5c). The stability between the biomaterials and the stacked scaffolds was maintained when exposed to heat (5 minutes at 200 °C) and mechanical stress (turning and flipping) during frying in oil (40 g oil; Fig. 5c). After frying, we observed a 14% reduction in height and a 27% reduction in weight (*n* = 1; Fig. 5d).

To investigate the impact of stacking on mouthfeel characteristics during chewing, scaffolds manufactured using two different approaches (printing and stacking) were analysed regarding their texture profile (Fig. 5h). Texture profile analysis was conducted and the hardness (N), cohesiveness, springiness and chewiness (N) were calculated from the data. The scaffolds, which had a layer thickness of 0.3 mm, were either screen printed three-dimensionally onto each other or printed individually and subsequently stacked. The texture profile after cooking was also analysed to compare raw and cooked scaffolds (Fig. 5h). The texture profiles of all variants (printed and stacked) were within the range of conventional meat, as shown by the values in the literature. Before cooking, the printed scaffolds were 40% softer than the stacked ones, as indicated by lower hardness values (*P* = 0.00231). After cooking, the stacked scaffolds became 94% softer (*P* = 0.0006), while the printed ones became only 19% softer. Cohesiveness and springiness were similarly high for printed and stacked scaffolds before and after cooking. Only significant differences were observed between raw printed scaffolds and raw stacked and cooked printed scaffolds (*P* = 0.01158; *P* = 0.02831). This consequently reduced the chewiness of the raw printed scaffolds by 26%. The other variants followed the same trend as the hardness.

### 3D bio-screen printed scaffold characterization

The microstructure, like the porous network of a material, impacts its physical properties. We therefore investigated different parameters to draw a conclusion about characteristics like the swelling and degradation behaviour.

Scanning electron microscopy was used to analyse the microstructure of the scaffolds to determine whether differences could be observed between the three-dimensional screen printing variants (mesh-based and stencil-based printing) (Fig. 5f, g). Scaffolds printed using stencil-based screens were found to have a porous structure similar to that of cast gels (Fig. 5f). The average area of open pores (porosity) was almost 50% for both (*P* = 0.91913; Fig. 5g). In comparison, scaffolds printed using mesh-based screens had a denser network of smaller pores (Fig. 5f). Porosity was significantly lower at 30% compared to stencil-printed and cast scaffolds (*P* = 0.00893; *P* = 0.01144; Fig. 5g). It can be seen for all scaffolds that the porous network consists of flat pores with an additional spiky structure, indicating interaction between the soy protein isolate and alginate materials (Fig. 5f). Furthermore, small, rounded accumulations probably represent salt deposits, such as calcium chloride and sodium chloride (Fig. 5f). Also, the surface of the scaffolds showed porous structure as can be seen in Supplementary Fig. 5.

The liquid absorption of 3D screen-printed scaffolds was investigated using two different screen variants (mesh-based and stencil-based). All lyophilised scaffold variants, including the casted control, exhibited a liquid absorption greater than a 1.5-fold increase (Fig. 5i). On average, stencil-printed scaffolds had a 3.6-fold change, which was not significantly different from the 4.4-fold change of casted gels (*P* = 0.1001). Mesh-based printed scaffolds had a significantly lower liquid absorption than stencil-based and casted scaffolds (1.8-fold change; *P* = 0.00106; 0.00005). Additionally, screen-printed scaffolds exhibited more consistent swelling (liquid absorption) compared to casted scaffolds. Wet layers that were crosslinked directly after screen printing showed a 0.15-fold change in liquid absorption and a 0.31-fold change when sterilised at 120 °C and then soaking in growth media (Supplementary Fig. 9).

Mass loss was analysed as an indicator of the degradation behaviour of the three aforementioned scaffold variants (Fig. 5j). Mesh-based printed scaffolds had a mass loss of 19.5%, which was significantly lower than that of cast scaffolds (20%; *P* = 0.96525). In comparison, stencil-based printed scaffolds had a mass loss of 10%, which was significantly lower than for mesh-based printed and cast scaffolds (*P* = 0.00078; *P* = 0.00055). Furthermore, as with swelling, printed scaffolds exhibited more consistent degradation behaviour (mass loss) compared to casted scaffolds.

**Fig. 6:**
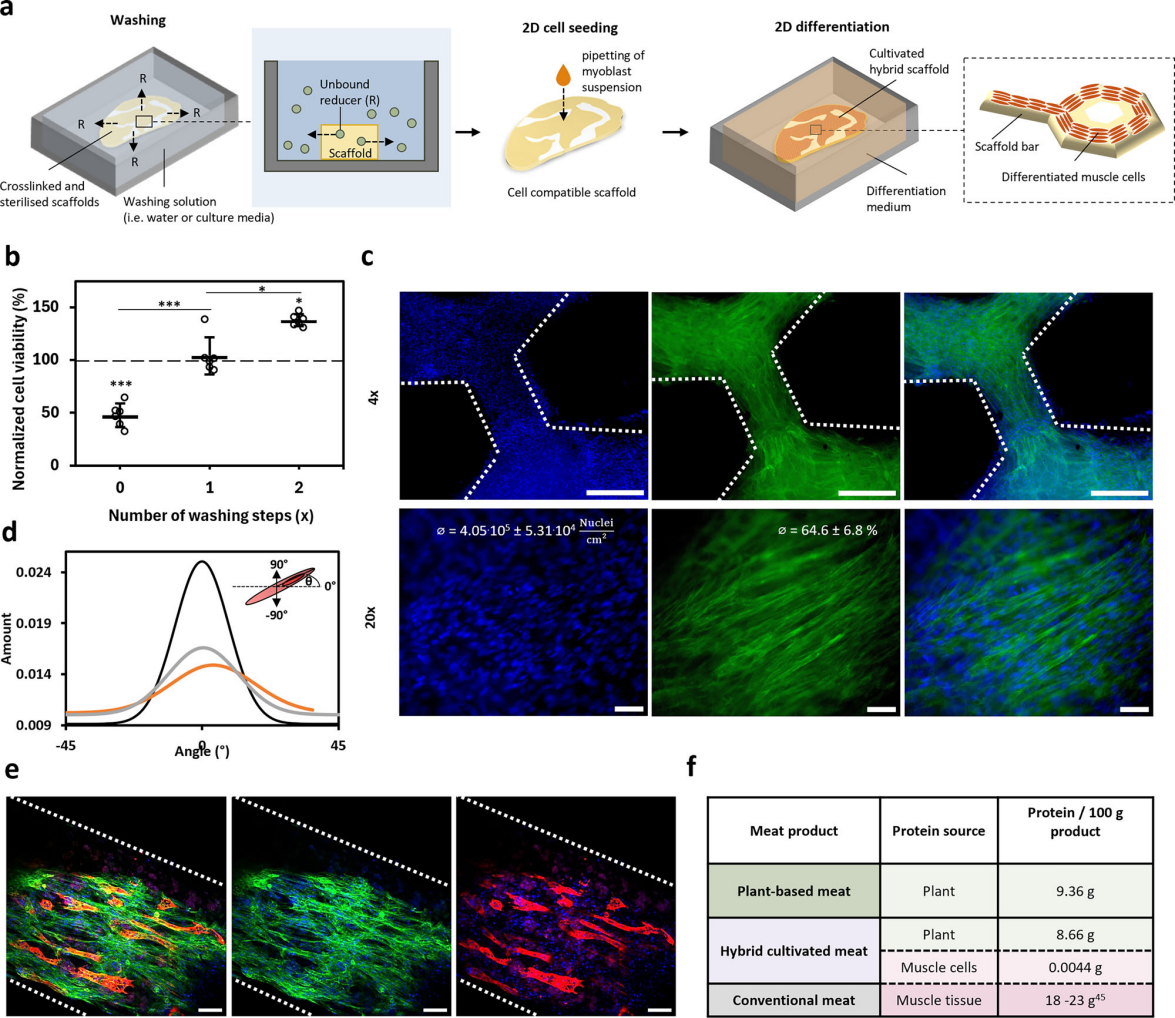
Compatibility of 3D bio-screen printed scaffolds for 2D myoblast differentiation. **a** The washing step from the overall process is shown to produce hybrid cultured meat using 3D screen-printed steak-like scaffolds in combination with 2D cell seeding. **b** Cell viability normalized to control over the number of washing steps (0x, 1x, and 2x) applied to reduced (sodium sulphite) SPI-scaffolds prior to 2D cultivation. Control represents treatment media without scaffold incubation. Horizontal lines show the mean, and error bars the standard deviation (SD), while single data points represent technical replicates with *n* = 6. A two-way ANOVA and a post-hoc Tukey test were used to compare data between washing repetitions. The Dunnett test was used to compare the data to the respective control. The significance levels are indicated as follows: *p < 0.05, **p < 0.005, ***p < 0.001. Asterisks with lines denote significant differences between groups and without lines to the control. **c** Microscopy images of actin (green) and nucleus (blue) staining after 2D cell seeding of C2C12 myoblasts onto 3D bio-screen printed scaffolds and 7 days of differentiation (4x and 20x magnification). Dotted lines represent edges of scaffold structures. Values represent the mean of nucleus density (left) and actin coverage (right). Scale bar = 50 µm for 4x and 500 µm for 20x. **d** Histogram of cell alignment determined from actin staining. Individual curves each represent a technical replicate. **e** Confocal images of actin (green), nucleus (blue), and myosin heavy chain (red) staining after 14 days of differentiation of C2C12 myoblasts on 3D bio-screen printed scaffolds. Dotted lines represent edges of scaffold structures. **f** Protein content determined from the plant-based and hybrid scaffolds was calculated per 100 g of meat substitute product, assuming that the protein concentration of the scaffolds and cells, as well as the cell density on the scaffold, remain proportional. In addition, a comparison with values from the literature of conventional meat is shown^45^.

The toxicity of reducing agents was tested due to concerns regarding the viability of cells during cultivation on scaffolds (Fig. 6b and Supplementary Fig. 6). Reducing agent concentration was determined experimentally towards optimal preparation and printing of the scaffolds (5% Na_2_SO_3_/15 g SPI). Besides Na_2_SO_3,_ the toxicity of cysteine (6.6%/15 g SPI) and glutathion (6.6%/15 g SPI) was tested as alternative reducing agents, with natural occurrence in cellular systems^44^. A concentration of 0.5% was required for cysteine (101 ± 22%, *P* = 0.223) and 0.1% for Na_2_SO_3_ (101 ± 8%, *P* = 0.00205) to

achieve high cell viability compared to the untreated control (Supplementary Fig. 6). 1% cysteine and 0.5% Na_2_SO_3_ significantly reduced cell viability to 36 ± 13% and 37 ± 3% (*P* = 0.00079; *P* = 0.0000000002; Supplementary Fig. 6).

Glutathione showed a constant level of viability over 70% without significant difference within the concentration range (0.01% and 0.005%, *P* = 0.989; Supplementary Fig. 6). All three reducers achieved high cell viability compared to the control at the lowest concentration applied here (0.005%; Cysteine, *P* = 0.9397; Na_2_SO_3_, *P* = 0.01441; Glutathione, *P* = 0.05479; Supplementary Fig. 6). We assumed that unbound reducer molecules were responsible for the decrease in cell viabilities. Repetitive washing steps were tested on the printed scaffolds to wash out the remaining reducer (Fig. 6b). For the washing protocol, reduced scaffolds (SPI with Na_2_SO_3_) were incubated in a 100 mM CaCl2 solution for different repetitions (0x–2x). Subsequently, the scaffolds were incubated in culture media, which were then used for C2C12 cell cultivation. Without prior washing steps (0x) the cell viability remained in mean lower than 50% in relation to the control (*P* = 0.00031167; Fig. 6b). After 1x washing, cell viability was on average as high as the control (104 ± 18%, *P* = 0.95631) and significantly higher when 2x washed (138 ± 6%, *P* = 0.00606; Fig. 6b). Even though sodium sulphite has a food number, the Joint FAO/WHO Expert Committee on Food Additives Dietary Sulphites recommends not exceeding a daily intake of 0-0.7 mg sulphites per body weight. We assumed that the washing protocol would significantly reduce the sulphite concentration in the scaffolds. To verify this, we measured the sulphite content in our scaffolds after washing. In Supplementary Fig. 8a, the sulphite content extrapolated to 1 kg of scaffold can be seen. As assumed, the sulphite content decreased with increasing washing steps. While an unwashed scaffold contains an average content of 1115 mg per kg of scaffold, this is significantly reduced to 49 mg sulphite/kg of scaffold when washed 2x. In order to estimate the consumption of such scaffolds, we calculated the values for daily intake and compared them to the maximum accepted daily intake (ADI) of 0.7 mg sulphite/kg body weight (Supplementary Fig. 8b). For this purpose, we assumed a meat consumption of 137 g per day, and a body weight of 70 kg. The values show that after washing twice, only 14% of the ADI remains in the scaffolds. This means that they would be safe to consume in terms of sulphite content. It should be emphasized again that our washing protocol was tested on a laboratory scale. Scaling up to an industrial scale, therefore, still needs to be validated. However, this also means that more efficient methods can be tested to reduce the sulphite content. Furthermore, this study presented alternatives to sodium sulphite (cysteine and glutathione) that may be more suitable in terms of consumption.

The 3D bio-screen printed plant-based scaffolds were investigated for 2D cell growth and differentiation of C2C12 myoblasts (Fig. 6c–e). A mean nucleus density of 4.05.10^5^ ± 5.31^.^10^4^ nuclei/cm^2^ and cell coverage of 64.6 ± 6.8% indicated a confluent cell population after 7 days in culture (Fig. 6c). Cells showed an elongated morphology (Fig. 6c) and high accumulation of oriented cells along a common axis (Fig. 6c, d). Myotube formation was observed after 14 days of differentiation, evident by myosin heavy chain expression. The myotubes had an elongated shape aligned along the long axis of the bar structures (Fig. 6e).

The total protein content of the hybrid scaffolds (which were seeded with cells) was examined. In addition, the protein content of the plant-based scaffolds (without seeded cells) was evaluated as a reference (Fig. 6f and Supplementary Fig. 7). The soy protein isolate used in this study contains approximately 90% protein. Therefore, the initial protein content of the screen printing ink was ~13.5% (w/w). The average total protein content of the plant-based and hybrid scaffolds was 65% and 60% of the original protein content, respectively (Supplementary Fig. 7). Furthermore, the total protein content of the hybrid scaffolds was found to be 7% lower than that of the plant-based scaffolds (*P* = 0.43315). The total protein content of myoblast cells proliferated and differentiated on the scaffold surface averaged 6.8 µg per cm² of scaffold surface area (Supplementary Fig. 7). To estimate the nutritional value of the scaffolds as a meat alternative, we calculated the protein content of a 100 g product, assuming that the protein concentration of the scaffolds and cells and the cell density on the scaffold would remain proportional (Fig. 6f). We determined a protein content of 8–10 g per 100 g of product and 4.4 mg of muscle cell protein. For comparison, the protein content of conventional beef is 18–23 g/100 g^45^ (Fig. 6f).

### Spatial complexity is enhanced by 3D cell culture with alginate as a binding agent

**Fig. 7:**
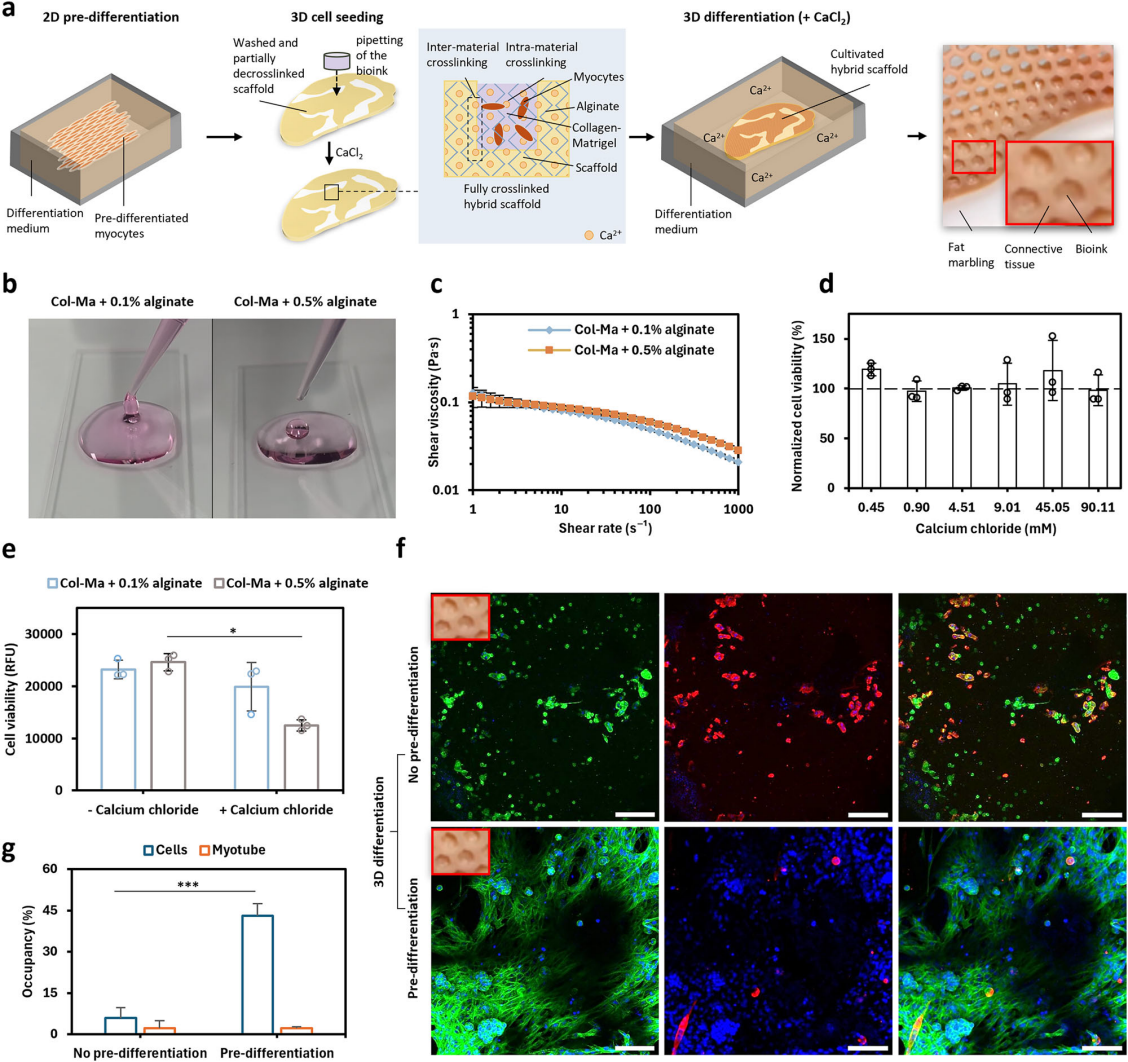
A strategy for 3D cell growth and differentiation in 3D bio-screen-printed scaffolds. **a** Necessary process steps for 3D cell cultivation in steak-like scaffolds for the production of hybrid cultivated meat from the overall process. **b** Photographic image of the pipetting of two Collagen-Matrigel-Alginate hydrogel formulations with different alginate fractions (0.1% and 0.5%). **c** Shear viscosity curves of the two Collagen-Matrigel-Alginate hydrogel formulations. **d** Normalized cell viability of 2D C_2_C_12_ cultures at different CaCl_2_ concentrations (0.45 mM–90.11 mM). Data were normalized to cell culture media without CaCl_2_. **e** C_2_C_12_ cell viability represented by relative fluorescence units for two Collagen-Matrigel-Alginate hydrogel formulations with different alginate fractions cultivated in media with or without CaCl_2_ (50 mM). A Tukey test was used to compare the data within an alginate fraction or coverage type. **f** Confocal images showing in top view fluorescence staining of myoblast cultures in scaffolds with and without 2D pre-differentiation taken from a day 7 culture. Actin (green), nucleus (blue), and myosin heavy chain (red) staining was applied for qualitative and quantitative analysis. Scale bar = 500 µm. **g** Bar charts of cell occupancy (blue) and myotube occupancy (red) along the z-axis taken from confocal images on day 7. A two-sample t-test was used to compare the data within a coverage type. The significance levels are indicated as follows: *p < 0.05, **p < 0.005, ***p<0.001. Asterisks with a line indicate significant differences between the groups. Dots and columns show the mean and error bars the standard error (SE) while single data points represent technical replicates with *n* = 3.

3D cell culture in scaffolds was investigated to mimic the three-dimensional nature of the native muscle tissue more reliably (Fig. 7a). A collagen and Matrigel blend based on the work of the Post lab^46^ was developed to ensure myoblast attachment to the hydrogel. Alginate was added for crosslinking between the bioink and the scaffold materials using CaCl_2_ (Fig. 7a). Two alginate concentrations (0.1% and 0.5%) were tested for this. The viscosity of both formulations was found to be suitable for pipetting into the open cavities of the scaffold (Fig. 7b, c). Myoblast viability was examined in 2D to confirm the compatibility of CaCl_2_ in cell culture media. No significant difference to the non-treated control was observed for the lowest (0.45 mM, *P* = 0.25593) and highest concentration (90.11 mM, *P* = 0.99756) applied here (Fig. 7d). In 3D culture, cell viability was not significantly decreased when 50 mM CaCl_2_ was added to the 0.1% bioink formulation (*P* = 0.07577; Fig. 7e). In comparison, cell viability decreased significantly when 50 mM CaCl_2_ was added the 0.5% bioink formulation (*P* = 0.03068; Fig. 7e).

To increase the differentiation efficiency in 3D cell cultures inside the scaffolds, we adapted a pre-differentiation protocol from Messmer et al.^46^ (Fig. 7a). After 7 days of 3D differentiation inside the scaffold cavities, we observed cell development that was found to be dependent on pre-differentiation in 2D before seeding the bioink into the scaffold cavities (Fig. 7f, g). Cell coverage was 7.41-fold higher with pre-differentiation compared to samples without pre-differentiation (*P* = 0.00039; Fig. 7g). Furthermore, both myocytes and myotubes exhibited an elongated morphology through pre-differentiation, while without pre-differentiation, we observed a rather round-shaped morphology (Fig. 7f).

## Discussion

Scaling up the biofabrication processes is a crucial step in the transition from laboratory to industrial-scale production of cultivated structured meat. In addition to current challenges such as the development of animal-free cell culture media^1^ and scaling of bioprocesses^47^, the biofabrication of structured meat represents an additional scaling dimension^14^. In this context, two challenges in particular need to be addressed: Current 3D bioprinting technologies are limited in their suitability for large-scale manufacturing of (hybrid) cultivated meat^14,48^ and the printing of high-protein materials at high resolution^18,23,25,27^ and high production capacity. In this study, we set out to develop a process that addresses the scalability limitations of current bioprinting technologies. The new biofabrication process, 3D bio-screen printing, has the potential to be scaled up to an industrial level for use in the large-scale production of structured cultivated meat. The process is based on traditional 2D screen printing and comprises the development of high-protein scaffold materials, the generation of meat-like structures, as well as 2D and 3D cell cultivation strategies with marbled scaffolds.

Soy protein isolate (SPI) has proven to be highly compatible for the production of scaffolds in 3D bio-screen printing. This is in particular due to its flow behaviour. At high protein content (12% - 21% in this study), it exhibits a high viscosity but also a strong shearing behaviour. This makes it very suitable for printing. The next step is to test the suitability of a wide range of materials, such as peas, field beans, and gluten. It can already be assumed that these materials will also be possible to process, as the viscosity range is similar, as well as flow index values are low for protein-based materials. In addition, textured plant-based protein could be printed to increase the fibrous texture of the scaffold towards conventional meat. The viscosity of the SPI-based scaffold material was in the range of traditional screen printing inks^31,49^. Shear thinning was sufficient within the shear rate window of screen printing (500–10,000 s^−^^1^) for complete material transfer onto the substrate. In order to initiate material flow, the yield point of the material must be overcome by the shear rate of the flodder and the squeegee. Although the yield point was not determined in this study, it can be assumed that it was low enough for the printing parameters used here (shear rate and shear stress). However, future studies should consider investigating the yield point. The gel-like behaviour of SPI resulted assumably from high intra-molecular entanglement driven by disulfide bonds, hydrogen bonds, hydrophobic effects, and other secondary bonding. This is also known to increase at higher SPI concentrations^26,30,50^. Technologies such as high moisture extrusion require high temperatures to process protein-rich materials. The temperatures can have a negative impact on the nutritional properties of the material. In addition, the need for high temperatures to melt the material is an energy-intensive process and, therefore, less environmentally friendly. A more energy-efficient approach was presented here by using reducing agents. Na_2_SO_3_ as a reducing agent prevented disulfide bonding, leading to higher flow capacity and lower viscosity. In our study, the addition of a reducing agent at 15% SPI led to a pronounced increase in the phase angle, indicating a higher viscous contribution and a weaker elastic network. This effect was much stronger than at 12% and 18% SPI. One possible explanation is that 15% SPI represents a transition region between network formation and protein aggregation. While at 12% SPI, a relatively mobile protein network can still form an elastic gel, increased aggregation occurs at 15%. Reduction of disulfide bonds likely prevents the formation of a stable cross-linked network, resulting instead in looser aggregates that dissipate mechanical energy more strongly, which is reflected in the higher phase angle. At 18% SPI, the protein concentration may be sufficiently high for physical interactions, such as hydrophobic interactions and steric crowding, to promote the formation of a denser structure despite reduced disulfide crosslinking. Additionally, the reduction of disulfide bonds may have shifted the percolation threshold of the protein network, which has been reported in the literature for SPI at around 7–8% protein^51,52^. Reducing agents with hydroxy groups (i.e., ascorbic acid or urea) might help to prevent gel formation at higher SPI contents^26,30^. In addition, alginate can be suitable as a thickening material to improve the printability and shape fidelity without impacting the flow behaviour. Finally, as a plant-based hydrogel, alginate can be sourced sustainably, underlining its suitability potential for the future of CM fabrication.

We tested printability using connective tissue-like structures as an example. To do this, fine bar structures measuring 0.1 m to 1 mm were printed. These formed cavities in various geometries and diameters, which were filled with bioink during our study in order to differentiate myoblasts into fibrous structures. In further applications and studies, the cavities could be filled with other materials, such as textured plant proteins. In addition, instead of cavities, column structures can be printed that directly mimic the fibrous structure of muscles. This would theoretically be possible with a resolution of 20 µm^53^.

Meat-like structures with a wall thickness of ≥ 100 µm were successfully 3D bio-screen printed without a reduction in throughput. Each layer was 15 µm high, and a volume shrinkage of about 50% along the build direction was assumed after curing. Each printing step took 4 seconds, while the curing time was 10 seconds, which is the time-limiting factor. For scaling, the curing time can be equalised to 0 s by transporting and printing several print tables in series via a conveyor belt. To cure the layers, we used hot air. In the future, a combination of hot air and infrared heating could be tested for more efficient curing. A further scaling step would be the usage of stencil-based screen printing to increase the height of the individual layers to 200 µm or more. However, it should be noted that the wall thicknesses achievable to date with a stencil screen are ≥ 200 µm. In addition, thicker layers may require longer curing times. The image accuracy (the absolute deviation of the printed structure from the intended printing image of the screen), and the reproducibility (standard deviation of the image accuracy) showed a high consistency along the applied resolution range. As in accordance with 2D screen printing, no trade-off exists between resolution and throughput in 3D bio-screen printing (3D-BSP)^32,36,37,49^. This indicates that with 3D-BSP, high production volumes (>100 kg/h) might be achievable at uncompromised accuracy in future applications, provided that the process can be adapted to industrial screen printing parameters. However, the curing method also has an influence on the measures of the printed structures. The measured deviations in the printed structures were therefore probably caused by material shrinkage, which is a normal effect during printing^54^. In addition to the hot air used here, as is used for industrial screen printing, infra-red emitters should be researched for more efficient and gentle curing. Larger structures had higher accuracy and showed a material deficit, while small structures experienced a material surplus. However, smaller structures are relatively more affected (dimensional non-linearity). The mesh opening used here had a diameter of half the size of the highest lateral resolution (0.1 mm/50 µm). A larger ratio (i.e., 0.1 mm/10 µm) can increase the print accuracy^36,37^. However, this requires smaller threat diameters and thus comes with lower layer heights^32^, which can impact the scalability^36^. Finally, deviation can affect cell growth and differentiation, texture, and nutritional values^17,55,56^. Therefore, adjustment of 3D bio-screen printing parameters is a critical step.

In principle, any 2D design can be recreated into its three-dimensional construct using 3D-BSP. A future approach, following on from the free design of the steak-like scaffold used here, may be to digitise the microscopic and macroscopic structure of conventional meat for screen manufacturing. This would allow to reproduce the natural appearance of meat more accurately. Structured CM requires the reproduction of muscle and fat tissue. In the present study, a plant-based biomaterial formulation was used for fat marbling, as the co-cultivation of muscle and fat cells is a complex process that has been little researched to date^57–59^. Nevertheless, cultivated fat offers a more reliable alternative to plant-based fat, which could be a future replacement. One strategy for its integration into structured CM is a co-culture of myo- and adipocytes on scaffolds^3,58,60,61^. In addition, adipocytes could replace the plant-based fat material used here or be embedded into it. The fat marbling can be applied either by one-step moulding using stencil printing or in layers. While the moulding approach offers a higher production speed, the layered application allows a better spatial distribution along the z-axis, which is advantageous for a more accurate imitation of conventional meat. The increasing elastic proportion at lower temperatures of the fat material can help with greater stabilisation after printing. Alginate ensured the intra-material stability of the scaffold biomaterials (connective tissue and fat material) during cell culture by ionic cross-linking. In addition, alginate was applied as a binding agent to ionically couple these two materials with different chemical compositions together (water-based SPI solution and soybean oil-based fat). For its long-term stability during cell culture, the addition of 50 mM CaCl_2_ to the cell culture media probably counteracted sodium, thus preventing decrosslinking^57,62^. The generation of tissue on a centimetre scale is currently limited by nutrient diffusion. Common strategies to circumvent this problem are the integration of blood vessels^58,63–65^ or the creation of cavities, pores or channels using sacrificial materials^66^. In this context, the here applied stacking approach of parallelly cultivated scaffolds might be a practical way to overcome the diffusion limit in 3D cell culture^58,64,67^. Before stacking, the short exposure to EDTA assumably reversed crosslinking of the outer layer of the scaffold material while retaining crosslinking of the inner lying material. Recrosslinking leads to successful binding of the individually printed scaffolds. Furthermore, the ioinic binding could also be kept stable during food preparation (pan-frying with oil). Future research into the bioprocesses of scaffolds must investigate how the stacking approach can be scaled up. To this end, it is conceivable that completely new bioreactor concepts will be created that automate both the parallel cultivation of cells on scaffolds and approaches such as stacking matured scaffolds or tissue sheets. In addition, the ingredients used in the cell culture medium, such as FBS, must be replaced by animal-free and sustainable alternatives^46,68–70^. In order to produce growth factors, the largest cost factor^68^, cost-effectively and sustainably, approaches such as recombinant expression in algae are interesting and should be researched further^71,72^. The weight loss of the scaffold is in accordance with conventional meat for the applied cooking method^73,74^. Height loss along the z-axis was lower than the longitudinal shrinkage of conventional meat. This is acceptable, since shrinkage in conventional meat is, among other things, caused by a decrease in muscle fibre length^75^.

The texture profile analysis was used to evaluate the chewing experience of the scaffolds. All cooked scaffolds had a texture profile that was within or close to the range of values reported in the literature for conventional meat^61,76–81^. While conventional meat becomes harder during cooking^76,77,80^, the scaffolds in this study became softer (lower hardness) after cooking, which was also shown in other studies^9^. In our study, entrapped air in the scaffolds expanding during cooking could be one reason for our observation. The high cohesiveness of the stacked scaffolds remained after cooking, showing that this approach can be used to retain the structural integrity of the scaffolds. The scaffolds were more elastic than conventional meat^78,79,82^, as shown by their higher springiness. This could be due to the scaffolds having a denser protein network, as well as the alginate stabilising the network further. Lowering the protein or alginate concentration or testing different crosslinking parameters could help to adjust the springiness further. Other crosslinking reagents, such as transglutaminase, could also be tested^83^. In terms of chewiness, printed scaffolds could provide a similar chewing experience to conventional meat^61,76,77,80^. For stacked scaffolds, testing parameters such as EDTA concentration and de- and recrosslinking might increase the chewiness to a level similar to that of conventional meat.

Based on the images of the microstructure, it can be assumed that crosslinking occurred between the soy protein isolate and the alginate^83–85^. The alginate is probably stabilising the hydrogel network and preventing it from dissolving in the cell culture medium. Larger pores may be caused by air entrapment during the stirring of the material preparation step. The difference in pore network between the two screen types (mesh-based and stencil-based) is probably due to heat treatment and higher squeegee pressure in combination with a lower printed layer thickness in the mesh-based approach. During mesh-based 3D screen printing, the application of temperature might have collapsed the pores, while the printing pressure of the thin layers compressed them. Since larger pores can improve nutritional support for cells seeded on scaffolds^16,86,87^, adapting the aforementioned parameters for the mesh-based approach should be investigated further.

The higher consistency in liquid absorption of printed scaffolds compared to cast scaffolds is likely due to the higher reproducibility of the printed layers. It can be assumed that the higher liquid absorption of stencil-based printed scaffolds compared to mesh-based printed scaffolds is due to their pore structure, as larger pores can absorb more water^87^. This can also generally lead to higher degradation, which makes the higher mass loss of mesh-based printed scaffolds seem contradictory. Since degradation also occurs on the surface of scaffolds^87^, topographical differences could be the reason for this. These surface differences could be due to the higher curing temperatures and higher squeegee pressure used in mesh-based printing. For all scaffolds, the mass loss could be the result of destabilization of the protein network after swelling in the growth medium. In other studies, a critical concentration of < 13% was determined for stable soy protein isolate gels^88^. As experiments on total protein content in our study have shown, the protein concentration drops below 13% during incubation in the growth medium.

The reducing agent used (Na_2_SO_3_) proved to be necessary at higher protein contents in the SPI-based ink in order to ensure the processability (flooding step) of the biomaterial. Reducing agents are commonly used as antioxidants in the food industry and cell culture^30,44,89,90^. Liver and kidney cells in particular can metabolize the sulphites to sulfates by the mitochondrial enzyme sulphite oxidase^91^. However, it is still considered harmful to specific individuals^92^. Furthermore, too high concentrations can cause oxidative stress to cells in vitro^92,93^. The concentration of 0.75% Na_2_SO_3_ (750 mg/100 g scaffold material) used here was higher than the concentration determined safe for consumption by the JECFA (0.7 mg/kg body weight, Joint FAO/WHO Expert Committee on Food Additives Dietary sulphites). However, after washing the scaffolds, the sulphite content in the scaffolds could be adjusted to the acceptable daily intake. This means that the scaffolds would be safe to consume in terms of sulphite content. It should be emphasized again that our washing protocol was tested on a laboratory scale. Scaling up to an industrial scale, therefore, still needs to be validated. However, this also means that more efficient methods can be tested to reduce the sulphite content. Nishiuchi et al. have shown a cell toxic cysteine concentration of 1 mM in culture media^94^. We observed nontoxic concentrations of ≤ 0.8 mM (0.1%), which seems to correlate with their findings. However, it must be considered that the culture media and cell type were different from our conditions. Furthermore, 5 mM pyruvate in the culture media or prior incubation at 37 °C for 24 h reduced cytotoxicity^94^, which represents another interesting approach for the here shown process. Glutathione is synthesized intracellularly by all mammalian cell types. An externally induced overdose might therefore disturb the physiological cell metabolism^90^. Remnants of the reducer after scaffold-washing probably acted as antioxidants, leading to relatively higher cell viability^44,90^. A reducer concentration greater than 0.1% is reasonable for non-washed scaffolds. Unbound reducing agent molecules inside the scaffold were probably flushed out and diffused into the media during incubation^29,95^, lowering the concentration inside the scaffold to less than 0.1%. 1x washing appears to be sufficient for maintaining cell viability, while 2x washing might introduce antioxidant effects of remaining reducer^44,90^.

Providing a solid surface with structural binding sites for cell adhesion is necessary for cell growth and differentiation^8,70,96^. The scaffolds produced using the 3D-BSP process presented here seem to fulfil this requirement. The SEM images show that the surface is not smooth but porous and has sinusoidal elevations. This surface pattern occurs in the printing process immediately after printing and usually smooths out until the layer has dried. Due to the rapid drying of the individual layers in our process, the sinusoidal structure may have been preserved. This structure could have promoted cell adhesion. The here observed cell alignment on the scaffold was thereby promoted in the bar region^17,55,56,97^. However, the degree of myotube formation determined in this study was comparably lower than that reported in in-vitro systems^46,98^. Myotube development depends on many factors, including material properties^8^, media formulation^16,46,70^, surface modification^15,16^, scaffold architecture^16–18,55,56^, scaffold stiffness^8,61^ and external stimulation^17,56,98,99^, whose optimization was not the focus of this work. Nevertheless, achieving a higher degree requires, among others, optimization of those parameters. The 2D differentiation of myoblasts on edible scaffolds is one way to improve nutritional value and sensory properties such as taste. This could also contribute to the texture of the cultivated construct. However, this may currently be less efficient than obtaining differentiated cells from a bioreactor and mixing them with the scaffold material, as each scaffold approach may require a unique bioreactor system. Nevertheless, the differentiation of myoblasts relies on solid surfaces to which the cells can attach, often in combination with necessary coatings. The scaffold could be designed to replace conventional surfaces and thus be efficiently integrated into bioreactor systems, while eliminating the need for coatings, as the SPI surface of the scaffold shown here is sufficient for adhesion and differentiation.

The total protein content of the two scaffold types (plant-based and hybrid) was found to be lower than the initial protein content of the ink. The ink's protein content of around 13.5% probably became diluted during crosslinking in the CaCl₂ bath and cultivation in the growth medium. This is supported by the liquid absorption experiments conducted here, in which the weight of the scaffolds increased by 15% during crosslinking and by 31% during cell culture. Consequently, the protein content is estimated to have decreased by approximately 2.1 g and 4.21 g. This correlates more closely with the loss of protein content in the plant-based scaffold (loss = 2.8 g–5.1 g) and more distantly with the hybrid scaffold (loss = 3.96 g–5.65 g). Additionally, the difference in total protein content between the two scaffold types could be attributed to their different swelling behaviours, given that hybrid scaffolds contained more water before the measurement. In conventional meat, the total protein amount is 18–23%^45^. It consists of ~80–90% muscle fibres and ~and 10-20% connective tissue proteins such as collagen, while the rest is water^4^. Plant-based and hybrid scaffolds had only about half the protein content of conventional meat. In plant-based scaffolds, the protein content consisted of soy proteins, while in hybrid scaffolds, cell proteins contributed to the total protein amount. However, at a total of 0.05%, this was very low, raising the critical question of the extent to which cells contribute protein to hybrid scaffolds. The focus should perhaps be on the contribution to the sensory profile, such as taste and chewiness. The positive influence of cells on this has already been demonstrated in other studies^61^. In addition, efficient 3D cultivation systems of cells in gels will assumable increase the protein content. To achieve this, animal-free gel formulations and culture media must be developed, as well as efficient differentiation protocols.

In this study, alginate was used as a sustainable and edible binding agent to hold together the three main components: connective tissue, fat marbling, and bioink. Divalent ions are necessary for inter-material cross-linking. CaCl_2_ was used here, which cross-linked the materials together. However, CaCl_2_ has the disadvantage that studies have shown it to be bitter even in small quantities. Therefore, other binding agents or alternative cross-linkers should also be considered in the future. Alginate as a binding agent in bioinks ensured calcium chloride-dependent crosslinking to the scaffold and stability during cell culture. Based on the results of our study, CaCl_2_ as a counterpart to 50 mM sodium in the media can be considered uncritical, as the viability of C2C12 myoblasts was consistently high in the presence of up to 100 mM CaCl_2_. This was also consistent with other studies^18,57,100^. The significantly lower viability of the cells in cross-linked gels with a higher alginate concentration of 0.5 % could be related to an increase in the mechanical properties of the gel, such as the stiffness of the gel. This could have led to an inhibition of the penetration of nutrients and oxygen into the gel^62,101^. In addition, disturbances in the cell proportions and cytoskeletal structure could have been caused by calcium deposits in the gel^62^. Adjusting the calcium chloride concentration might help to adjust the mechanical properties, such as stiffness or pore size, of the gel and reduce deposits to increase viability^62,84^. Here, collagen and Matrigel were only used as demonstrators, which are known for their biofunctionality. In future studies, the formulation of bioink for myoblast culture must be replaced by plant-based hydrogels. Alginate-RGD in particular would be a promising candidate. It provides important peptide binding sites for cell adhesion and can also be used for the mechanisms of de- and re-crosslinking of bioinks in the scaffold presented here^10,18^. In this study, we investigated an approach to promote myoblast differentiation according to a protocol reported by Messmer et al.^46^. We were able to confirm that the 2D pre-differentiation of myoblasts promotes the growth and differentiation of muscle cells in the 3D environment.

In summary, this work demonstrates the potential of 3D bio-screen printing (3D BSP) as a biofabrication process that could be suitable for large-scale production of cultivated meat. The process shows that, with successful transfer of industrial parameters from traditional industrial screen printing, comparatively high production volumes could be achieved without significantly compromising the print resolution, precision, or basic functionality of myoblast cultures on printed scaffolds. With regard to future applications in the food sector, the results suggest that 3D-BSP may be capable of processing protein-rich materials into structures with high resolution (0.1 mm) on an industrial scale. In this context, the study presents a first use of reducing agents in biofabrication, which could offer new possibilities for material processing. The proposed stacking process, based on the reversible de- and re-crosslinking of alginate, offers a potential route to the creation of centimetre-scale scaffolds that could help overcome diffusion-related limitations. Under the conditions tested, the printed scaffolds supported both 2D and 3D myoblast culture and differentiation. In addition, a marbled demonstrator was produced that includes areas mimicking muscle and fat. Due to ionic interactions between the compartments, the demonstrator remained intact during a basic step of food preparation (pan frying). Overall, the individual methods explored in this study appear promising for the development of future optimization strategies in cultivated meat research. Furthermore, 3D-BSP could be relevant beyond this field and potentially adapted for other biofabrication applications, such as bioprinting for medical research (e.g., organ-on-a-chip systems or tissue models), warranting further investigation.

## Methods

### Scaffold material preparation

Deionized water, sodium sulphite (5% of SPI, Sigma Aldrich), alginate (1%, sodium Alginate from brown algae, Sigma Aldrich, Taufkirchen, Germany), and SPI (12%, 15%, 18% or 21%, MP Biomedicals) were mixed (% w/w). The solution was stirred in a 50 °C water bath with a high-speed stirrer (Heidolph, Hei-Torque 200) at 1200 rpm for 10 min. The complete material was directly used for further experiments. For the fat substitute (FS), 3% (% w/w) sodium alginate powder was dissolved in deionized water at 70 °C. Soybean oil (SO) was separately heated to 70 °C. SPI was dissolved in the heated alginate solution (1%/SO (% w/w)). The SO was then added to the alginate-SPI solution. The mixture was thoroughly stirred until a creamy texture and a homogeneous emulsion were achieved. The FS was used immediately for further experiments.

### Rheological measurements

A rotating, oscillating rheometer (Netzsch, Panalytical Kinexus lab+) was used for all rheological measurements. For shear viscosity, a plate-plate setup was used with an upper plate of 1° cone and 40 mm diameter. The measurement was carried out at 1 Pa, 1 Hz, and 25 °C. The flow index value "n" and the consistency factor “K” were calculated using the Ostwald-de Waele model. For oscillatory measurements, the temperature was decreased from 45 °C to 4 °C, using a flat PU40 geometry at 1 Hz and 1 Pa.

### Flooding coverage efficiency

First, 100 g of the SPI solutions (12%, 15%, 18% and 21% with and without 5% Na_2_SO_3_/SPI) were cast on a satin glass substrate (Glastechnik Rhein-Main, Darmstadt, Germany). Next, the material was flooded using a coating machine (Zehntner, ZAA 2300) at a velocity of 10 mm/s. For imaging, a Canon camera and light setup (Walimex pro, Soft LED 200 Flat Bi Colour) were used. Images were analyzed using ImageJ Fiji with a maxEntropy threshold to determine the uncovered area. The coverage efficiency was calculated as the percentage of the total area covered.

### Cytotoxicity of reducing agents and CaCl_2_

Cell titre blue (CTB) assays were performed to quantify the cell viability of C2C12 cells. C2C12 cells were cultivated in a microtiter plate with a cell density of 3300 cells/cm^2^. The cells were then incubated for 48 hours at 37 °C and 5% CO_2_. After 48 hours the medium was replaced by growth media with the corresponding concentration of sodium sulphite (Sigma Aldrich), L-Cysteine (Sigma Aldrich), glutathione (0% (control) 0.005, 0.01, 0.05, 0.1, 0.5 and 1% v/v) or CaCl_2_ (0.9001, 04505, 0.0901, 0.0451, 0.0090 and 0.0045 mM) and incubated for another 24 hours at 37 °C and 5% CO_2_. Growth medium was used for the positive control and the blank value. After 24 hours, the medium was replaced by the CTB solution (80 µl growth medium and 20 µl CTB reagent) and incubated for two hours at 37 °C and 5% CO_2_. The CTB solution was analysed using a plate-reading fluorometer (Tecan, Infinite 200 pro microplate reader) at respective excitation and emission (λ = 560 and λ = 590).

### Washing protocol for scaffolds

For washing, autoclaved scaffolds with a weight of 0.1 g were incubated in 3ml of a sterile washing solution (100 mM CaCl_2_, Sigma Aldrich) for 5 min at RT. After the respective number of washing steps (0x, 1x and 2x), the scaffolds were incubated for 1 hour at 37 °C in growth media supplemented with 50 mM CaCl_2_, resulting in the treatment solutions. The no-washing step (0x) was incubated directly in growth media without prior washing steps. The treatment solutions were added to the cells according to the CTB assay as described above.

### 3D bio-screen printing of plant based scaffolds

The printing images for the scaffolds were first designed in 3D using Fusion 360 and then two-dimensionalised using Inkscape. Mesh-based screens were manufactured by Hans Frintrup GmbH (Germany). Screen parameters were as follows: metal frame, covered with a stainless-steel mesh with a fineness of SD 50/30 (50 threads per cm/30 µm thread thickness), an angle of 22.5° and 15 µm EOM (emulsion over mesh). A waterproof photosensitive emulsion was used as a mask. For stencil printing, stencils were laser-cut from an aluminium sheet with a material thickness of 0.3 mm (Christian Koenen GmbH, Germany). 3D bio-screen printing was conducted using an in-house developed automatic printing machine on the basis of a semi-automatic laboratory screen printing machine (Rokuprint, SD05). The machine was technically extended by a microcontroller-based system to print automatically in 3D through the control of the number of layers, screen offset, pressure control, and curing system. Satin glass was used as a substrate (Glastechnik Rhein-Main, Darmstadt, Germany). For printing the scaffolds, 100 g of the SPI-R-A scaffold material was applied to the screens and printed until the required scaffold height was reached. For mesh-based printing, the following parameters were used: round squeegee with a hardness of 63 shore A, squeegee angle of 10°, squeegee pressure on the substrate of 6539 Pa, an aluminium flooder with a gap to the screen of 1 mm, 100% process speed (0.5 mm/s) and screen offset of 1.6 mm. Printed layers were heated by a hot air dryer (Leister, Hotwind System) during the whole printing process, for which the temperature was set to 70 °C with 400 litres/min applied air volume (level 3). The distance to the layers was about 50 cm. For the fat substitute (FS), 100 g material was applied to the stencil and printed into the 3D scaffold using a round squeegee with a hardness of 63 shore A, a squeegee angle of 60°, squeegee pressure on the substrate of 131 Pa, 10% process speed (0.15 m/s), and a screen offset of 0 mm. For stencil-printed scaffolds, the same parameters as for the fat substitute were used.

A 3D laser scanning microscope (VR-5200 3D profilometer, Keyence, Japan) was used to image the scaffold structures in automatic mode at 40x magnification. Multifile Analyzer software (Keyence, Japan) was used to analyze the profile and thickness of the structures. The profile was measured using the profile function and the thickness was measured using the plane measurement function. For bar and cavity diameters, three structures of each geometry were measured and the statistical values of the boxplot were calculated from the totality of all geometries.

### Texture profile measurement

For the TPA measurement, two variants of scaffolds were produced, both with a height of 0.9 mm. The first variant consisted of three-dimensional scaffolds printed on top of each other in layers. The second variant consisted of individual printed scaffolds that were stacked to the final height. For both variants, stencil printing and SPI-A scaffold material were used. A stencil with a material thickness of 0.3 mm and a rectangular printing form was used. The same screen printing parameters were used as for fat marbling. For the stacking approach, individual scaffolds with a wet layer thickness of 0.3 mm were printed. For the three-dimensionally printed scaffolds, three layers were printed on top of each other per scaffold with a wet layer thickness of 0.3 mm each. For this purpose, the screen offset was increased by 0.3 mm per layer. After printing the scaffolds, they were cross-linked with 100 mM CaCl2 for 1 hour. The scaffolds for the stacking approach were then washed in deionized water and incubated in 100 mM EDTA for 5 min to de-crosslink the outer material areas. The scaffolds were then air-dried. Three scaffolds were stacked on top of each other and re-crosslinked in 100 mM CaCl2. Both the stacked and three-dimensionally printed scaffolds were incubated in CaCl2 for a further 24 hours to ensure complete crosslinking of all samples. After 24 hours, the scaffolds were autoclaved. For the pressure force measurements of cooked samples, printed and stacked scaffolds were fried in a pan with oil at 200 °C for 1 minute on each side. All pressure force measurements were performed on a pressure-tension machine (ZwickRoell Z050) with a 50 N force transducer. Circular samples were prepared from the scaffold for the measurements. For this purpose, discs were punched out of the stacked and three-dimensionally printed scaffolds using a 10 mm hole punch. The TPA measurement consisted of a cyclic load with a number of two cycles. The measurement started at 0.05 N preload and a speed of 0.05 mm/s. The sample was then loaded at a loading speed of 3 mm/s to a load point of 50% and then unloaded at the same speed. After a holding time of 1 s at the unloading point, the sample was reloaded according to the first cycle. The hardness (N), cohesiveness, springiness, and chewiness (N) were calculated from the measured values. For hardness, the maximum of the first load was taken; for cohesiveness, the area of the load curve of the second cycle was divided by that of the first cycle. For springiness, the effective compression path of the second cycle was divided by that of the first cycle. Chewiness was calculated as the product of hardness (N), cohesiveness, and springiness.

### Microstructure of the scaffolds

To analyse the microstructure of the scaffolds dependent on the screen type, mesh-based scaffolds were printed using a mesh-based screen with the above-described parameters from the printability experiment. Stencil-based scaffolds were printed using a stencil-based screen with the parameters described above from the TPA experiment. For the casted samples, some material was taken from the scaffold ink with a spoon. All scaffolds were crosslinked using 100 mM CaCl₂ for 1 hour and subsequently sterilised at 120 °C for 30 minutes. Next, the scaffolds were freeze-dried (Alpha 1-2 LD plus, Christ). The scaffolds were sputtered with argon (Cressington Sputter Coater 208 HR), and the microstructure was imaged using scanning electron microscopy (SEM), for which a Zeiss EVO 10 SEM was used under vacuum at a voltage of 10 kV.

### Liquid absorption of the scaffolds

Two independent experiments were conducted to investigate the swelling behaviour of screen-printed scaffolds. First, the effect of crosslinking and cell cultivation conditions on the swelling of autoclaved scaffolds was analysed. Second, the effect of the screen type on swelling was analysed. For crosslinking-dependent swelling, the scaffolds were printed on glass slides using stencil printing with parameters from the TPA experiment, and the weight was measured directly (W1crosslinked). Subsequently, the scaffolds were transferred to a 100 mM CaCl₂ bath and maintained at 37 °C for 24 hours. For the autoclaving experiment, the scaffolds were printed and crosslinked in the same way, and then autoclaved at 120 °C for 30 minutes. The weight of the scaffold was measured (W1 autoclaved) and then it was incubated in C2C12 growth medium at 37 °C for 24 hours. After 24 hours, the remaining liquid was removed from the scaffolds and their weight was measured again (W2_crosslinked_, W2_autoclaved_). For screen-type-dependent swelling, mesh-based and stencil-based scaffolds were printed using a mesh-based screen with the above-described parameters. Stencil-based scaffolds were printed using a stencil-based screen with the parameters described above from the TPA experiment. For the casted samples, some material was taken from the scaffold ink with a spoon. All scaffolds were crosslinked using 100 mM CaCl₂ for 1 hour and subsequently autoclaved. Next, the scaffolds were lyophylised (Cressington sputter coater 208 HR), after which their weight was measured (W1_lyo_) using a precision scale. The scaffolds were then soaked in growth medium containing 50 mM CaCl₂ and maintained at 37 °C for 24 h. The remaining liquid was then removed from the scaffolds, which were weighed again (W2_wet_). The liquid absorption for all samples was calculated as (W1 - W2)/W2.

### Mass loss of the scaffolds

To assess mass loss, the scaffolds were prepared as described above for liquid absorption, after which their weight was measured following the first lyophilisation (W1_lyo_). The scaffolds were incubated in C2C12 growth medium for seven days, with the medium changed every 48 hours. After seven days, the scaffolds were lyophilised again and the second weight was measured (W2lyo). Mass loss was calculated using the following equation: ((W1_lyo_ - W2_lyo_)/ W2_lyo_)* 100.

### Preparation of scaffolds for cell culture

After printing, scaffolds were crosslinked by placing them into a sterile-filtered CaCl_2_ (Sigma Aldrich) bath with a concentration of 100 mM with an incubation duration of 5 min at RT according to the washing protocol described above. This step was repeated twice to ensure the removal of free reducing agent. The scaffolds were then autoclaved at 120 °C to sterilize them and subsequently air-dried under the cell culture bench. To simplify handling during cell culture, the scaffold construct was reduced in size by punching out circular structures with a diameter of approximately 2.5 cm (cut-out moulds) containing representative portions of the scaffold and marbling.

### Cell culture

C2C12 myoblast cells (CLS Cell Lines Service GmbH, Eppelheim, Germany, passage 34) were cultivated at 37 °C and 5% CO_2_ in plasma-treated flasks (VWR), changing medium every 48 hours. Growth medium was DMEM high glucose (Gibco, Dulbecco’s Modified Eagle Medium (1x) [4.5 g/L D-Glucose, - pyruvate]), 10% FBS, 1% Pen/Strep (Invitrogen) and 1% Amphotericin. After reaching confluence, cells were harvested for cell viability assays or 2D seeding. For C2C12 pre-differentiation, growth medium was replaced with differentiation medium (DMEM high glucose, 2% Horse Serum, 1% Pen/Strep/AmphB) and differentiated for 48 hours in the flask, before being embedded into hydrogels.

### Bioink preparation

Bioinks were prepared as follows: collagen solutions were prepared at 4 °C, consisting of 1x PBS, 3 mg/ml collagen (6 mg/ml, Sigma Aldrich, Collagen solution from bovine skin) 0.01 M sodium hydroxide (Sigma Aldrich) and 0.395x medium. For a collagen Matrigel solution, the collagen solution was mixed with 7% Matrigel (Sigma Aldrich, Matrigel Matrix) at 4 °C. For a collagen Matrigel alginate solution, the collagen Matrigel solution was mixed with sterile filtered alginate to an end concentration of 0.1% or 0.5% alginate (sodium Alginate from brown algae, Sigma Aldrich, Taufkirchen, Germany) at 4 °C. Cell suspensions were then added and resuspended. Bioink was kept at 4 °C until pipetting to scaffolds, or into well plates as a control.

### Cell seeding on scaffolds

2D cultures and bioinks were manually seeded onto the scaffolds using a pipette (Eppendorf). 2D C_2_C_12_ cultures were seeded at a concentration of 100,000 cells/scaffold. The scaffolds were incubated for 1h at 37 °C and 5% CO_2_ to allow for cell attachment. Cells were cultivated in growth medium for 5 days, supplemented with 50 mM CaCl_2_ to prevent decrosslinking by counteracting monovalent cations. Next, cells were differentiated in differentiation media containing 50 mM CaCl_2_ for 9 days. For 3D systems, the bioink was seeded into the cavities of the cut-out moulds or into well plates (bulk gels) and incubated at 37 °C at 5% CO_2_ for 30 minutes to ensure gelation of the bioink. Finally, the cell-loaded scaffolds and bulk gels were cultivated in differentiation medium supplemented with 50 mM CaCl_2_ to allow crosslinking between the bioink and the scaffold. Cultivation with the same differentiation media at 37 °C and 5% CO_2_ was used until fixation. Media were changed every 48 hours.

### Fluorescent staining

For immunofluorescent staining, the samples were fixed in 4% formaldehyde (Rotifix) for 15 min. Next, permeabilization was done with 0.1% Triton-X in Ringer solution. Next, blocking solution (5% Goat-Serum in Ringer solution) was added for 30 min. A primary antibody (Invitrogen, anti-sarcomeric a-myosin heavy chain (a-MHC) antibody MF20, 1:500 dilution in 5% Goat-Serum in Ringer solution) was added and incubated overnight at 4 °C. A secondary antibody (Invitrogen, Goat anti-Mouse IgG2b Cross-Adsorbed Secondary Antibody, Alexa Fluor™ 594, 1:500 dilution together with Thermo Scientific, Phalloidin Alexa Fluor 4800, 1:400 dilution in 5% Goat-Serum in Ringer solution) was added and incubated for 1 h. The constructs were washed three times for 10 min with Ringer's solution. Next, DAPI (1:1000 dilution) was added. The constructs were in fresh Ringer solution for microscopy. Images were taken either with confocal microscopy (Leica TCS SP8” confocal microscope) with application of the maximum projection function or fluorescence microscope (Revolve, Echo, San Diego, CA) at 4x and 20x magnification. For 2D cell cultures, cell coverage, nucleus density, cell alignment and myotube coverage were determined from fluorescence microscope images (Revolve, Echo, San Diego, CA). Confocal microscopy images (Leica TCS SP8” confocal microscope) were used for 3D cell cultures. Analysis was conducted using ImageJ Fiji.

### Total protein isolation from cultivated scaffolds

First, scaffolds were printed using a rectangular printing mould with a thickness of 0.3 mm and the scaffold material formulation SPI-A (15% w/w). Three layers were printed three-dimensionally on top of each other at a squeegee speed of 0.15 mm/s and a squeegee angle of 30°. The scaffolds were then cross-linked for one hour in a 100 mM CaCl_2_ solution. The scaffolds were then sterilized for 30 minutes at 120 °C. The scaffolds were cut to a final area of 2 cm × 1.5 cm. C2C12 cells were seeded onto the scaffolds (hybrid scaffold) at a cell density of 16,666 cells/cm² (50,000 cells per scaffold). In addition, scaffolds without cells seeded onto the surface (plant based scaffold) were created as controls. The scaffolds were incubated for 1 hour at 37 °C and 5% CO_2_ to allow the cells to attach to the surface of the scaffolds. The scaffolds were then transferred to a new, non-surface-treated well plate to prevent cells from attaching to the surface of the wells. The scaffolds were incubated for three days in c2c12 growth medium (+50 mM CaCl2) and then for four days in differentiation medium (+50 mM CaCl2). After the 7-day culture period, the scaffolds underwent total protein isolation. For protein isolation, the scaffolds were incubated in TrypLE™ Express (Gibco) at 37 °C for 10 minutes. After rinsing the scaffold surface with the TrypLE solution, the solution was collected in a centrifuge tube. This was repeated three times until no visible cells were seen under the microscope. Next, the cells were centrifuged at 250 x *g* for 5 minutes. The supernatant was discarded and the pellet was washed by resuspending in PBS 1x after centrifugation. The pellet was then suspended in 100 µl RIPA Lysis Buffer (Thermo Scientific) for cell lysis and protein extraction. The cell lysis and extraction solution was placed on ice for 30 minutes and then centrifuged at 10,000 x *g* for 10 minutes. The supernatant containing the proteins was collected. The amount of protein was determined using the Pierce BCA protein assay (Thermo Scientific) according to the protocol. The plant scaffolds were removed from the culture medium and weighed. They were then transferred to a new vessel. 5 ml of a solution of 0.1 molar NaOH and 100 mM EDTA was added to the scaffolds and heated in a water bath at 95 °C for at least 30 minutes to dissolve the scaffolds. The scaffolds were stirred intermittently. Once all scaffolds had dissolved completely, they were cooled to room temperature and the pH was neutralized with HCl. The scaffolds were then centrifuged at 4000 x *g* for 5 min and the supernatant was transferred to a new tube. This contained the proteins. The supernatant was used in the BCA according to the protocol to determine the protein concentration.

### Sulphate content in washed scaffolds

First, scaffolds were printed using a rectangular printing mould with a thickness of 0.3 mm and the scaffold material formulation SPI-A (w/w% SPI). Three layers were printed on top of each other in three dimensions at a squeegee speed of 0.15 mm/s and a squeegee angle of 30°. The scaffolds were then cross-linked for one hour in a 100 mM CaCl2 solution. The scaffolds were then sterilized for 30 minutes at 120 °C. Before washing, each scaffold was weighed using a precision scale. The scaffolds were then washed according to the washing protocol described above, using fully deionized water as the washing solution. Scaffolds were prepared for the extraction of the sulphite content for analysis using a modified protocol from Robbins et al.^102^: First, the scaffolds were homogenized. To do this, 10 times their weight in 0.2% formaldehyde was added to the scaffolds and homogenized with a hand mortar. The homogenates were transferred to a tube and centrifuged in a rotator at 70 rpm for 10 minutes. The same volume of 0.2% formaldehyde was then added to the samples. Next, the samples were cooled in an ice bath and treated with ultrasound at 90% amplitude and 0.8 cycles. The samples were then centrifuged at 4000 x *g* for 5 minutes and the supernatant was transferred to a new tube. Next, the samples were purified using an SPI C18 column (Bond Elut C18 cartridge, 500 mg, 6 mL, 40 um, Polyethylene, Agilent Technologies). The second application of the sample from the column was collected and heated to 80 °C for 30 minutes. The sulphite content was measured at the Institute for Wastewater Technology (Technical University of Darmstadt) using liquid chromatography ion exchange. The samples were stored at 4 °C until measurement. Before measurement, the samples were adjusted to a pH value > 10 and left to stand for 2 hours to convert the sulphite into hydroxymethane sulfonate and formaldehyde. Next, a 37% H₂O₂ solution was added to the samples to oxidize the sulphite to sulphate. In addition to measuring the sulphate, the sulphite concentration was determined to ensure complete oxidation of the sulphites. Daily meat intake was calculated at 50 kg of meat per person per year/365 days. The acceptable daily intake (ADI) was calculated at 0.7 mg/kg body weight * 70 kg body weight of an adult.

### Statistical analysis

Statistical analysis of the experimental data was performed using Origin (OriginLab Corporation, Northampton, MA, USA) and MATLAB (MathWorks, Natick, MA, USA). Normal distribution was evaluated using the Shapiro-Wilk test or the Kolmogorov-Smirnov test, while variance homogeneity was tested using the Levene test. A parametric test (unrelated t-test or ANOVA) was performed for normally distributed and equal variance data. Non-parametric tests (Wilcoxon test, Kruskal-Wallis test or Mann-Whitney test) were used for non-normally distributed or variance-unequal data. For post-hoc comparisons between groups, the Tukey test (parametric) or the Dunn test (non-parametric) was used. A significance level of *p* < 0.05 was considered statistically significant.

## Supplementary information

Supplementary Information

## Data Availability

The data supporting the findings of this study were generated internally only and are available upon request from the corresponding author.
