# Author Correction: 3D bio-screen printing for high-throughput production of scaffolds for meat alternatives

**DOI:** 10.1038/s41538-026-00927-z

**Published:** 2026-06-11

**Authors:** Robin Maatz, Philipp Karnop, Ryan Sylvia, Thomas Herget, Andreas Blaeser

**Affiliations:** 1 Institute for BioMedical Printing Technology, TU Darmstadt, Darmstadt, Germany; 2 Merck KGaA, Darmstadt, Germany; 3 Centre for Synthetic Biology, TU Darmstadt, Darmstadt, Germany

**Keywords:** Biotechnology, Engineering, Materials science

Correction to: *npj Science of Food* 10.1038/s41538-026-00853-0, published online 13 May 2026

In this article, the wrong figure appeared as Fig. 6. The original article has been corrected.

