# Supplementary material for: 3D bio-screen printing for high-throughput production of scaffolds for meat alternatives
Source: NPJ Sci Food. 2026 May 13;10:155. doi: 10.1038/s41538-026-00853-0 (PMC13172316; doi:10.1038/s41538-026-00853-0)
Supplement: Supplementary file 1 — Supplementary Information [file 41538_2026_853_MOESM1_ESM.pdf]

Supplementary information

3D bio-screen printing for high-throughput production of scaffolds for meat alternatives

Robin Maatz[1]*, *Philipp Karnop*[1], *Ryan Sylvia*[2], *Thomas Herget*[1,2], *Andreas Blaeser*[1,3]*

[1]Institute for BioMedical Printing Technology, TU Darmstadt, Darmstadt, Germany,

[2]Merck KGaA, Darmstadt, Germany,

[3]Centre for Synthetic Biology, TU Darmstadt, Darmstadt, Germany

**Correspondence to: blaeser@idd.tu-darmstadt.de or maatz@idd.tu-darmstadt.de*

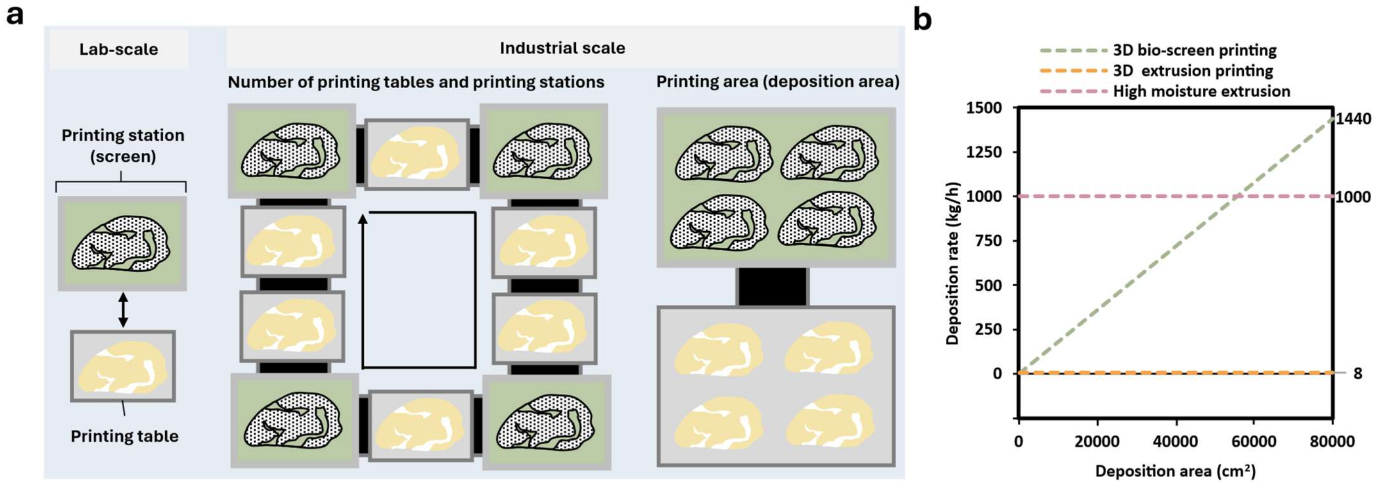

Supplementary Fig. 1 | Upscaling of 3D bio-screen printing for industrial production of meat alternatives. **a,** Scaling parameters for 3D bio-screen printing for the perspective transfer from laboratory to industrial scale. **b,** Anticipated production capacity in 3D bio-screen printing by larger printing areas. The values were calculated assuming a continuous printing with layer heights of 200 µm and a printing time of 4 seconds per layer. The calculation presumes that the parameters of industrial screen printing are applicable to 3D bio-screen printing. The data is compared with 3D extrusion printing and high moisture extrusion, both of which have a constant throughput that does not increase with the area to be deposited. The values were taken from the literature and industry reports. Four 3D extrusion printers with a nozzle size of 0.8 mm and deposition rate of 2 kg/h were used for the calculation.

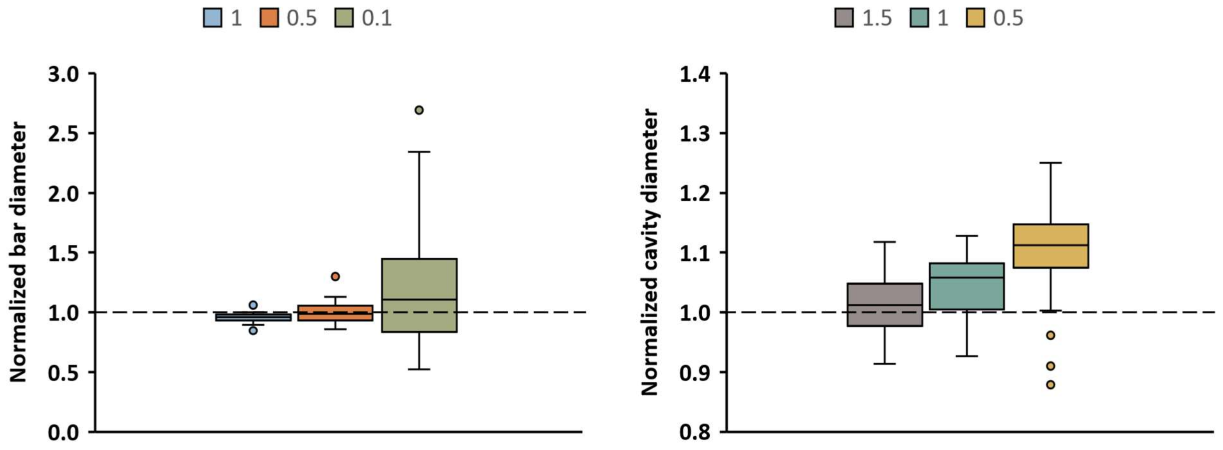

Supplementary Fig. 2 | Printability of 3D bio-screen printing of connective tissue like structures. Boxplots for normalized bar diameter (left) and normalized cavity diameter (right) calculated from the totality of all structures with n = 27 for bar diameters and n = 54 for cavity diameters. Data were normalized to the diameter of the theoretical design, represented as scattered line.

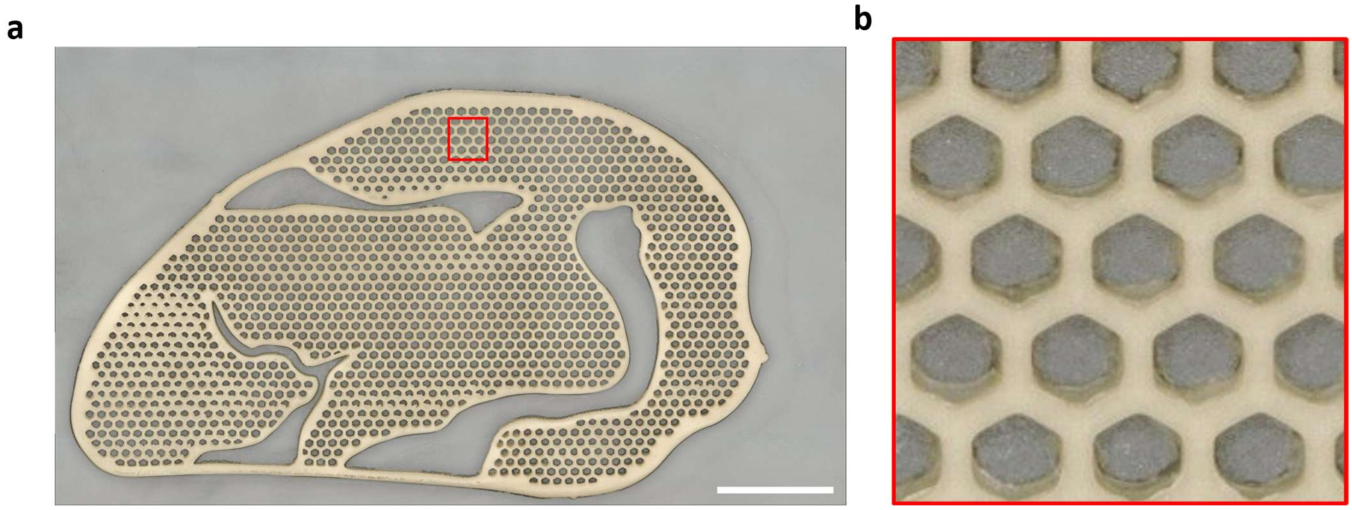

Supplementary Fig. 3 | 3D bio-screen printing of a marbled hybrid cultivated meat scaffolds. a, Microscopic image of the printed connective tissue part of the scaffold with **b,** close-up view of the hexagonal cavities.

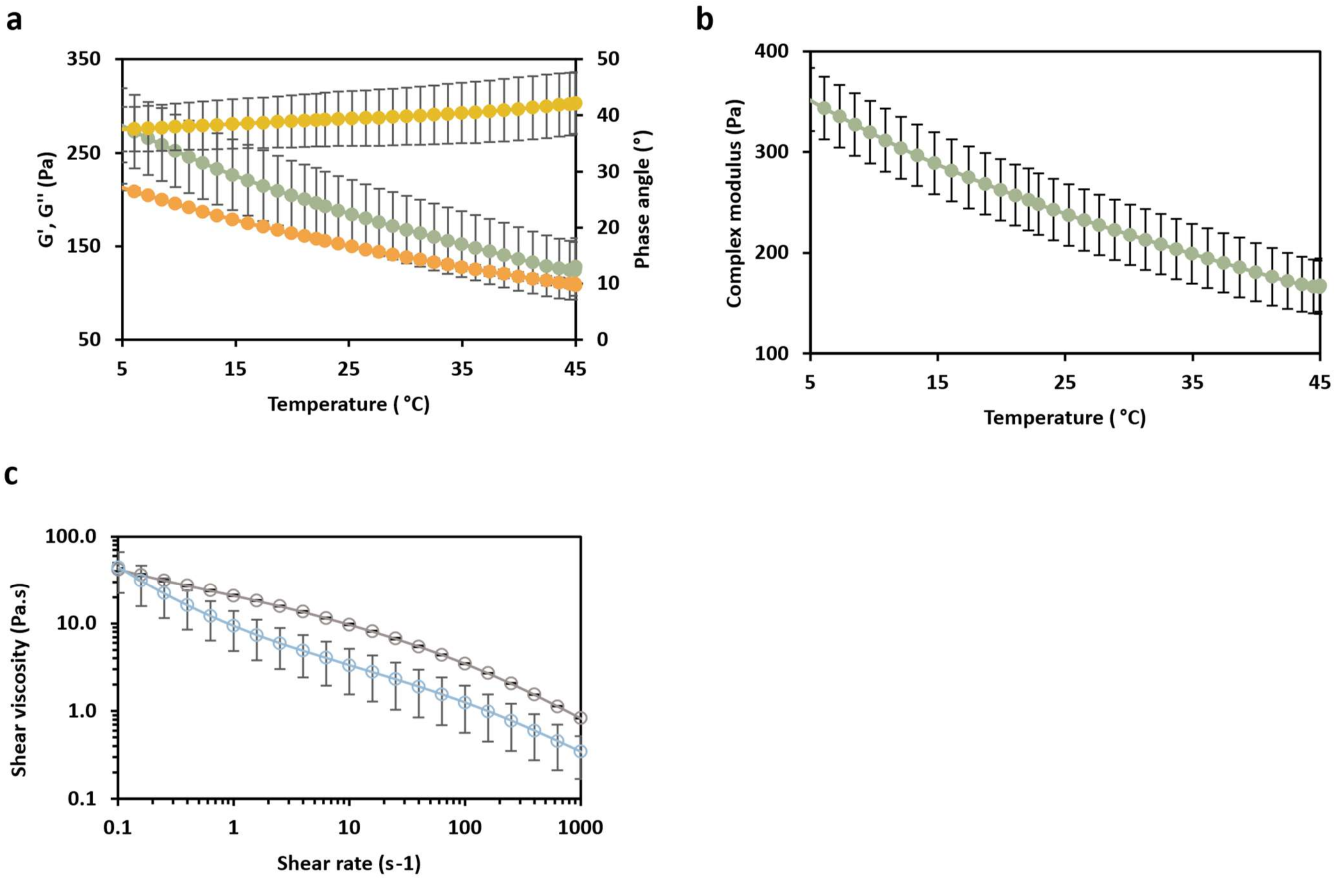

Supplementary Fig. 4 | Viscoelastic properties of the fat substitute. a, Elastic (G', green) viscous modulus (G'', orange) and phase angel (ɸ, yellow) over decreasing temperature (45°C - 5°C). **b,** Complex modulus (G*) of the fat marbling material for decreasing temperature (45°C - 5°C). **c,** Shear viscosity (η) of the fat marbling material, with curves for increasing (grey) and decreasing shear rates (blue)**.** Data points represent the mean, and error bars indicate the standard deviation with n = 3.

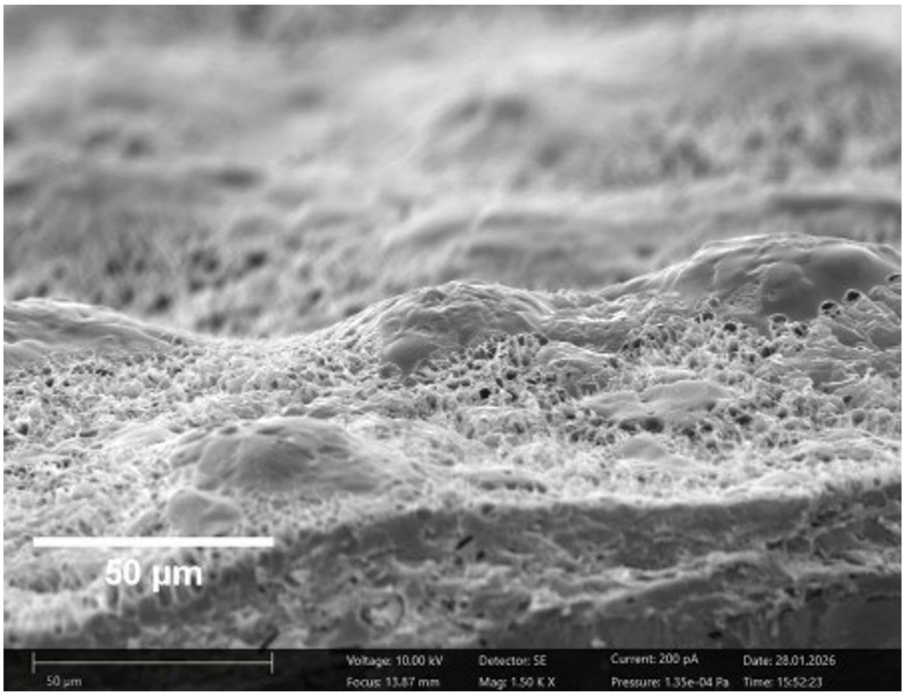

Supplementary Fig. 5 | Surface morphology of mesh based printed scaffolds. Scatter electron microscopy (SEM) image of mesh based printed scaffolds.

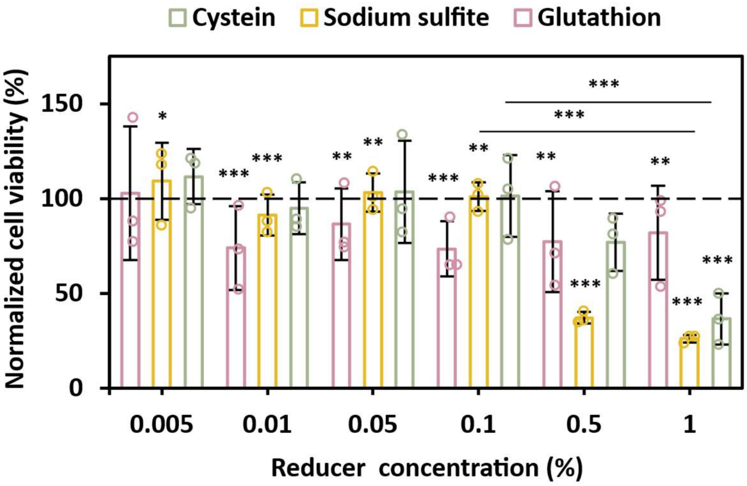

Supplementary Fig. 6 | Concentration dependent cell compatibility of reducing agents. Cell viability normalized to untreated control (no reducer) for different reducing agent concentration of cysteine (green), sodium sulphite (yellow) and glutathione (pink). Columns represent the mean and error bars the standard error (SE) while single data points represent technical replicates with n = 3. A two-way ANOVA and a post-hoc tukey test was used to compare data within a reducer type. Dunnett test was used to compare the data to the respective control. The significance levels are indicated as follows: *$p<0.05$, **$p<0.005$, ***$p<0.001$. Asterisks with lines denote significant differences between groups and without lines to the control.

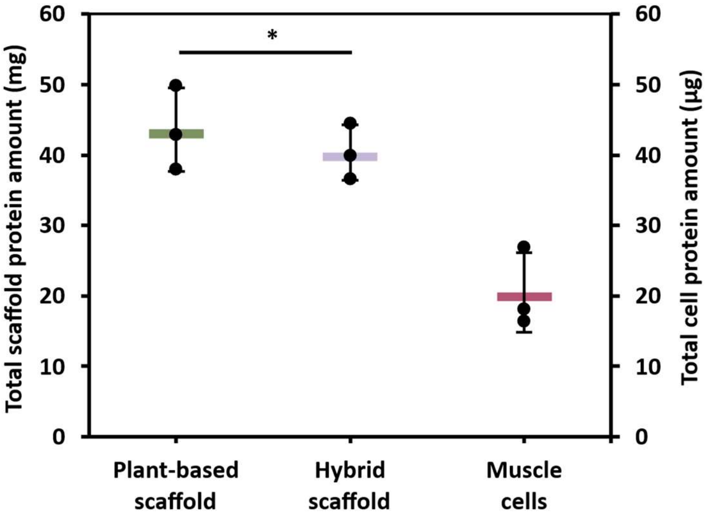

Supplementary Fig. 7 | Protein amount in plant based and hybrid scaffolds. Total protein amount from 0.5 g plant and hybrid scaffolds (left y-axis, in mg) as well as from cells seeded on hybrid scaffolds (right y-axis, in µg). The horizontal lines show the mean, the error bars show the standard deviation (SD), and the individual data points represent technical replicates with n = 3. A two-tailed t-test was used to compare the data between the two groups. The significance level is indicated as $*p < 0.05$.

a

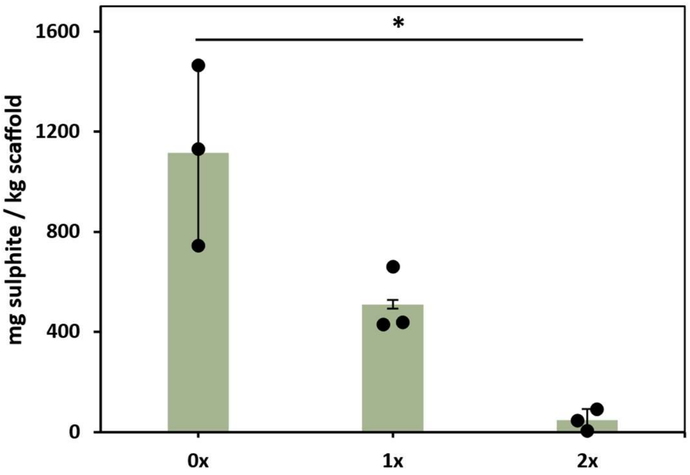

b

Washing step	Sulphite / daily meat intake (137 g)	% of ADI 70 kg (49 mg)
0x	153 mg	312 %
1x	70 mg	143 %
2x	7 mg	14 %

Supplementary Fig. 8 | Sulphite concentration in scaffolds after washing protocol. a, Sulphite concentration in printed scaffolds after 0x, 1x and 2x washing in water. The bars show the mean and the error bars show the standard deviation (SD), while the single data points represent technical replicates with n = 3. A two-way ANOVA and a post hoc Tukey test were used to compare the data across the three groups. The significance levels are indicated as $* p < 0.05$. **b,** Sulphite content of washed (0x, 1x, 3x) scaffolds in relation to the Acceptable Daily Intake (ADI) recommended by the Joint FAO/WHO Expert Committee on Food Additives for dietary sulphites.

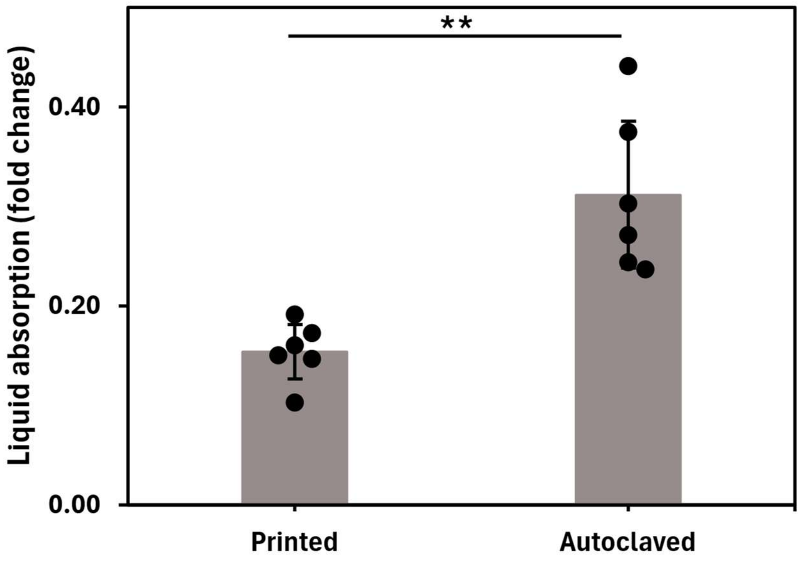

Supplementary Fig. 9 | Swelling behaviour for scaffolds after post-printing process steps. Liquid absorption of crosslinked wet layer directly after printing (printed) or after sterilisation at 120°C (autoclaved). Bars show the mean, and error bars show the standard deviation (SD), while single data points represent technical replicates with n = 6. A two-handed t test was used to compare the data of the two groups. The significance level is indicated as ** $p < 0.005$.
